# Subgingival microbiota in health compared to periodontitis and the influence of smoking

**DOI:** 10.3389/fmicb.2015.00119

**Published:** 2015-02-24

**Authors:** Anny J. Camelo-Castillo, Alex Mira, Alex Pico, Luigi Nibali, Brian Henderson, Nikolaos Donos, Inmaculada Tomás

**Affiliations:** ^1^Department of Health and Genomics, Centre for Advanced Research in Public Health, Fomento de la Investigación Sanitaria y Biomédica de la Comunitat Valenciana FoundationValencia, Spain; ^2^Oral Sciences Research Group, Special Needs Unit, School of Medicine and Dentistry, University of Santiago de CompostelaSantiago, Spain; ^3^Periodontology Unit, Department of Clinical Research, University College London-Eastman Dental InstituteLondon, UK; ^4^Department of Microbial Diseases, University College London-Eastman Dental InstituteLondon, UK

**Keywords:** 16S ribosomal RNA, chronic periodontitis, dental plaque, microbiome, oral health, smoking

## Abstract

The etiology of periodontitis has traditionally been associated to a consortium of three bacterial species—the so-called “red-complex” of periodontal disease—which has been the target for most diagnostic and therapeutic strategies. However, other species have also been found to correlate with disease severity. In addition, the influence of smoking on periodontal microbiota is poorly understood. In the current manuscript, the composition of the subgingival microbiota in healthy individuals vs. patients with chronic periodontitis has been investigated using 16S pyrosequencing and the influence of smoking on periodontal composition has been examined. Subgingival bacterial communities were sampled from 82 patients: 22 non-smoking healthy controls, 28 non-smoking periodontal patients, and 32 smoking periodontal patients. Bacterial diversity was higher in periodontal patients than in healthy subjects, which could be interpreted as the consequence of a nutritionally richer environment or a reduced immune competence. Periodontal patients showed a significantly higher prevalence/relative abundance of “established” periopathogens but also other taxa whose role is not well-established and that should be targets for future research. These include *Anaeroglobus*, *Bulleidia*, *Desulfobulbus*, *Filifactor*, *Mogibacterium*, *Phocaeicola*, *Schwartzia* or *TM7*. The microbial community of smoking-associated periodontitis is less diverse and distinct from that of non-smokers, indicating that smoking has an influence on periodontal ecology. Interestingly, the high sequencing coverage allowed the detection at low proportions of periodontal pathogens in all healthy individuals, indicating that chronic periodontitis cannot be strictly considered an infectious disease but the outcome of a polymicrobial dysbiosis, where changes in the proportions of microbial consortia trigger the inflammatory and tissue-degradation responses of the host.

## Introduction

The periodontal diseases are among the most common conditions affecting humans (Dentino et al., 2013). Periodontitis is a microbially-driven inflammatory condition of the gingivae causing destruction of the ligament and alveolar bone supporting the teeth resulting in oral malodor and tooth loss with the resultant loss of quality of life (Al-Harthi et al., 2013).

In 2012, data from the 2009 and 2010 National Health and Nutrition Examination Survey (NHANES) on the adult U.S. population showed that over 47% of adults had periodontitis, representing 64.7 million adults (Eke et al., 2012). Assuming this incidence of periodontitis worldwide, then 400 million people in Europe are suffering from one or other of the various forms of periodontitis.

In addition, there is starting to be convincing evidence that periodontitis might be a predisposing factor in the development of major systemic diseases such as coronary artery disease, cerebrovascular disease, diabetes, respiratory disease, and rheumatoid arthritis (Otomo-Corgel et al., 2012; Gulati et al., 2013).

Periodontal diseases are multifactorial diseases, whose initiation and progression require the participation of a number of factors, particularly the involvement of subgingival bacteria (Socransky et al., 1998). The idea of specificity in the bacterial etiology of periodontitis caused the scientific community to focus on the role of some putative periodontal pathogens such as *Porphyromonas gingivalis, Tannerella forsythia*, and *Treponema denticola* (Socransky et al., 1998). Page and Kornman (1997) expanded this model to acknowledge the contributions of genetic and environmental risk factors. Smoking is a well-established risk factor for periodontitis progression (Johannsen et al., 2014). However, there are conflicting reports, based on culture or targeted DNA-based assays, on whether or not smoking has an effect on the composition of the subgingival microbiota (Haffajee and Socransky, 2001; Van der Velden et al., 2003; Apatzidou et al., 2005; Darby et al., 2005).

In recent years, sequencing of cloned 16S rRNA genes has been widely used to analyze the subgingival bacterial composition and to characterize compositional shifts in periodontal health and disease (Paster et al., 2001; Kumar et al., 2005; Shchipkova et al., 2010). However, it has been demonstrated that next-generation sequencing (NGS) methodologies provide an economical and significantly higher-throughput alternative to the above for comparative genomics and identification of oral microbial communities (Siqueira et al., 2012; Nyvad et al., 2013). To date, few authors have compared the subgingival microbiota of periodontal patients to those of healthy individuals using pyrosequencing of 16S rRNA genes (Griffen et al., 2012; Liu et al., 2012; Abusleme et al., 2013; Li et al., 2014), and some of them using small sample sizes that may not be representative (Liu et al., 2012). Equally, there is scarce evidence on the influence of smoking habit on the subgingival microbiota using a NSG approach for bacterial identification (Bizzarro et al., 2013). It is therefore necessary to study more profoundly the microbial changes occurring in the periodontal pocket at the onset and progression of the disease, under the influence of risk factors such as smoking, confirming results from a statistical point of view. Thus, the aim of the present study was to use pyrosequencing of the16S RNA gene to define the composition of the subgingival microbiota in relatively large groups of periodontally-healthy non-smoking individuals with those of patients with chronic periodontitis who either are smokers or are non-smokers.

## Material and methods

### Selection of study groups

A total of 123 subjects, 72 affected by moderate to severe generalized chronic periodontitis and 51 periodontally healthy controls were recruited among consecutive patients who were referred to the Periodontology Unit of the School of Medicine and Dentistry (University of Santiago de Compostela, Spain) for an assessment of oral health status. Patients were selected if they fulfilled the following inclusion criteria: (i) age 30–65 years old, (ii) good general health, no pregnancy or breastfeeding, (iii) no intake of systemic antimicrobials during the previous 6 months, (iv) no intake of anti-inflammatory medication in the previous 4 months, (v) no routine use of oral antiseptics, (vi) no presence of implants or orthodontic appliances, (vii) no previous periodontal treatment, viii) presence of at least 18 natural teeth.

One experienced calibrated dentist performed all periodontal diagnosis. Probing pocket depth (PPD) and clinical attachment level (CAL) were recorded throughout the mouth on six sites per tooth using a PCP-UNC 15 probe. Bleeding on probing and bacterial plaque levels was recorded full mouth on a binary scale (presence/absence) on six sites per tooth (BOP and BPL, respectively).

Patients were diagnosed as suffering from moderate to severe generalized chronic periodontitis based on the previously established criteria (Armitage, 1999; Page and Eke, 2007). The control group included periodontally healthy individuals with no sites with PPD ≥4 mm, no radiographic evidence of alveolar bone loss and BOP <20%.

Smoking history was obtained using a questionnaire, collecting information on smoking status (never, past, or current), years (months) of smoking and number of cigarettes/day. All answers were reviewed with the subject by a member of the study team. In accordance with the smoking history, the final sample size was 82 patients and the distribution of the groups was as follows:

- Non-smoking healthy controls (*n* = 22; NS-Control).
- Non-smoking periodontal patients (*n* = 28; NS-Perio).
- Smoking periodontal patients (*n* = 32; S-Perio).

The recruitment of periodontally-healthy smokers was attempted, but during the collection period only six subjects were recruited. Therefore, it was decided to exclude this very small group of patients to avoid negative effects on the statistical analysis.

Patients who agreed to participate in the study signed a written informed consent. The protocol of the study was approved by the Ethics Committee of the Faculty of Medicine and Dentistry (University of Santiago de Compostela, Spain; 29/03/2011).

### Subgingival sample collection

Based on Simón-Soro et al. (2014), the subgingival plaque samples were collected at the same time of day (in the afternoon, approximately 5–7 h after toohbrushing). Samples were collected from each subject by inserting a total of 16 sterile endodontic paper points (size 30; two paper points per site) into the gingival sulci or periodontal pocket, for 10 s, following isolation and supragingival plaque removal.

Based on Simón-Soro et al. (2013), subgingival samples from periodontally healthy patients were collected by passing the paper points across each gingival sulci and pooled from eight teeth of the quadrants 1 and 3 (incisor, canine, premolar, and molar). In periodontal patients, subgingival samples were collected and pooled from the deepest PPD sites in each quadrant (a total of eight non-adjacent proximal sites).

Samples were placed in 1.5 ml microcentrifuge tubes with 300 μl of phosphate buffer frozen at −80°C until further analysis.

### Subgingival processing

#### DNA extraction, PCR amplification, and pyrosequencing

Before DNA extraction, bacteria were separated from the paper points by washing with 300 μl of phosphate buffer to the tubes and vortexing. DNA was extracted separately from each sample using the MasterPure Complete DNA and RNA Purification Kit (Epicentre Biotechnologies, Madison, WI, USA), following the manufacturer's instructions, with the addition of a lysozyme treatment (5 mg ml^−1^, at 37°C for 30 min) (Belda-Ferre et al., 2012).

PCR amplification of the 16S rRNA gene was performed with the high-fidelity ABGene DNA polymerase (Thermo Scientific) by the universal bacterial primers for the 16S RNA gene, 8F (5′-AGAGTTTGATCMTGGCTCAG-3′), and 533R (5′-GCTTACCGCGGCKGCTGGCACG-3′) using an annealing temperature of 52°C and 30 amplification cycles, following Simón-Soro et al. (2013). Two PCRs were performed per sample, pooling the two PCR products before purification. In 41 subgingival samples, a PCR product could not be obtained by this single-amplification approach and a nested-PCR was performed, in which the PCR product obtained after 20 cycles of primary amplification was purified with the Nucleofast PCR purification kit (Macherey-Nagel) and used as a template for a secondary PCR in which the primers were shifted 3 bp toward the 3′ end and modified to contain pyrosequencing lib-L adaptors A and B, following Benítez-Páez et al. (2013). This secondary PCR was performed for 20 cycles at an annealing temperature of 56°C to improve specificity.

The 500 bp PCR products were purified with the Nucleofast PCR purification kit (Macherey-Nagel) and further cleaned by AMPure XP beads (Roche) before pyrosequencing. The final DNA per sample was measured by picogreen fluorescence in a Modulus 9200 fluorimeter (Turner Biosystems) so samples could be mixed in equimolar amounts, adding 50 ng from each PCR product. PCR products were pyrosequenced from the forward primer end only by using a GS-FLX sequencer with Titanium-plus chemistry (Roche) at the Center for Public Health Research in Valencia, Spain. One-eighth of a plate was used for each pool of 25 samples, which were amplified with a different forward primer containing a unique 8-bp “barcode.” Barcoded primers allowed multiple amplified samples to be mixed in a single pyrosequencing reaction on eighths of a plate (Simón-Soro et al., 2013). The sequences obtained were deposited in the MG-RAST server under Accession Number ID 12161.

#### Data analysis

The sequences were separated using the “barcodes” and reads with an average quality value lower than 20 and/or with more than four ambiguities in homopolymeric regions in the first 360 flows were excluded from the analysis. Read ends were trimmed in 10 bp windows if they had an average quality value lower than 20, using the Galaxy tool (Blankenberg and Hillman-Jackson, 2014). Only reads longer than 250 bp were considered, as well as those without mismatches in the primer region. Chimeric sequences were detected using Mothur software v1.21.0 (Schloss et al., 2009) and 5% of reads were filtered out as potential chimeras. Singletons were not excluded from the analysis.

Rarefaction curves were calculated at the 97% sequence identity using the program CD-HIT (Li and Godzik, 2006) to produce operational taxonomic units (OTUs) at the species level (Sogin et al., 2006) and constructing the curves using the Analytic Rarefaction software (Holland, 2003). The Chao and Shannon indexes were used to calculate the total number of species-level phylotypes, normalizing samples to the same number of sequences by random sampling of the reads with the RDP pipeline (Cole et al., 2009).

Principal Coordinates Analysis (PCoA) was performed with Fast UniFrac using a weighted algorithm (Lozupone et al., 2006, 2007). Two-way comparisons in bacterial composition were performed using the UniFrac metric in order to measure whether the microbial communities in the different groups were significantly different. The sample identifications are randomly permuted 1000 times to obtain a *P*-value representing the fraction of permuted trees that have UniFrac values greater than or equal to that of the real tree, using the Bonferroni correction for multiple comparisons.

Sequences were taxonomically assigned using the Ribosomal Database Project classifier (Cole et al., 2009). Each read was taxonomically assigned down to the genus level using an 80% confidence threshold. For those genera that had significant differences in presence/relative abundance between healthy and diseased individuals, an attempt was done to assign the reads to the species taxonomic level. In order to do this, a curated database was constructed with the full-length sequences of all species belonging to the selected genera that were present in the Ribosomal Database Project. A BlastN sequence comparison (Altschul et al., 1997) was then performed against this database. The top hit from each sequence comparison was selected if the alignment length was >350 bp, and the sequence identity >97%.

All values from the quantitative variables related to clinical parameters presented variances which were not homogeneous, determined using the Levene test. For these variables, the Kruskal-Wallis and U Mann-Whitney non-parametric tests were used. Fisher's exact test was performed to analyze the variable “sex” between study groups.

A univariate statistical analysis was then performed to compare the prevalence of subjects with presence of genera between three study groups using Fisher's exact test; this test was also used for pairwise comparisons. The proportional means of abundance of genera between three study groups were analyzed using the Kruskal-Wallis non-parametric test and Mann-Whitney U-test for pairwise comparisons. The correlations between mean distributions of the more significant genera, and between these and clinical parameters were analyzed by Spearman Correlation Coefficient. The significance level applied was *p* < 0.05. The probability after multiple comparisons was also evaluated using Bonferroni correction, applying a significant level of *p* < 0.016. The results were analyzed using the PASW® Statistics Base 20 package for Windows (IBM, Madrid, Spain).

## Results

The analysis of clinical variables related to oral health status showed that, in comparison with the NS-Control, the periodontal groups had significantly higher values of BPL, BOP, PPD, and CAL (full mouth). At the microbial sampling site level, there were differences in BOP, PPD, and CAL between the three study groups, these being significantly higher in the periodontal patients than in the NS-Control (Table 1). The NS-Perio group showed significantly higher values of BOP than those observed in the S-Perio group. In the S-Perio group, the mean number of cigarettes/day was 15.9 and the mean number of months of smoking, 325.9 (Table 1).

**Table 1:** **Age, sex and clinical characteristics associated with the oral health status and smoking habit of the three study groups**.

**Clinical Parameters**	**Study Groups**				
	**NS-Control^a^ (I group; *n* = 22)**	**NS-Perio^a^ (II group; *n* = 28)**	**S-Perio^a^ (III group; *n* = 32)**	***P*-values**	***P*-values (intergroups)**
Age (years)	44.95 (13.04)	54.14 (11.90)	49.19 (7.46)	0.047	I–II^*^
**SEX**					
Male	8	8	16	NS	
Female	14	20	16		
Teeth (no.)	26.36 (3.51)	25.61 (4.67)	25.50 (4.00)	NS	
**FULL MOUTH**					
BPL (%)	22.43 (14.33)	53.29 (27.44)	54.00 (23.61)	<0.001	I–II; I–IIII
BOP (%)	13.86 (6.70)	57.98 (20.80)	43.56 (17.48)	<0.001	I–II; I–III; II–III
PPD (mm)	2.08 (0.30)	3.35 (0.64)	3.55 (0.79)	<0.001	I–II; I–III
CAL (mm)	2.25 (0.37)	3.98 (1.12)	4.35 (1.32)	<0.001	I–II; I–III
**SAMPLED SITES**					
BOP (%)	7.89 (12.64)	71.45 (25.59)	52.07 (24.51)	<0.001	I–II; I–III; II–III
PPD (mm)	2.35 (0.33)	5.84 (0.87)	5.86 (1.15)	<0.001	I–II; I–III
CAL (mm)	2.42 (0.36)	6.03 (1.02)	6.44 (1.61)	<0.001	I–II; I–III
Cigarettes/day (no.)	0.00 (0.00)	0.00 (0.00)	15.94 (8.14)	NA	
Months of smoking (no.)	0.00 (0.00)	0.00 (0.00)	325.93 (113.46)	NA	

*Values are means (standard deviations) and numbers of subjects*.

*NS-Control, Group of non-smoker healthy controls; NS-Perio, Group of non-smoker periodontal patients; S-Perio, Group of smoker periodontal patients; BPL, bacterial plaque level; BOP, bleeding on probing; PPD, probing pocket depth; CAL, clinical attachment level*.

a *Patients were diagnosed as periodontally healthy individuals or patients with moderate to severe generalized chronic periodontitis based on the previously established criteria*.

*A patient was defined as a smoker if he/she was currently smoking and had been smoker for at least 8 years and as a non-smoker if he/she had never smoked or quit smoking longer than 5 years before the sampling*.

* *After Bonferroni correction for multiple analyses (the significance level applied was p < 0.016), differences were no longer significant for these comparisons*.

### Subgingival microbiota in health and periodontitis: influence of smoking habit

#### Bacterial diversity and community structure

**Figure 1:**
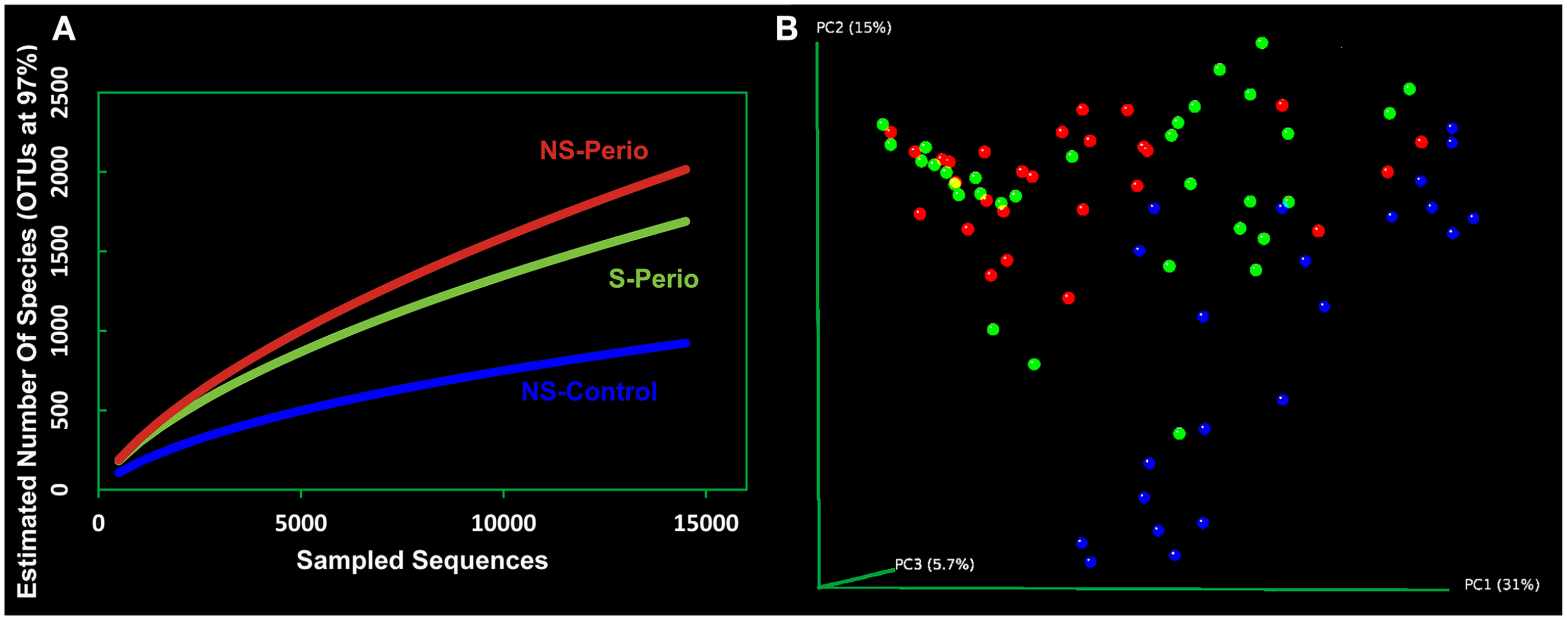
**Bacterial diversity and community structure. (A)** Rarefaction curves of all the subgingival samples by study group. The horizontal axis shows the number of sequences obtained by pyrosequencing the 16S gene. The vertical axis shows the number of operational taxonomic units (OTUs) at a level of 97% intersequence similarity, representing an approximation to the number of species for the sequencing effort employed. **(B)** Principal Coordinates Analysis (PCoA) of all 82 subgingival samples according to bacterial composition, including NS-Control (blue dots), NS-Perio (red), and S-Perio (green); PCoAs were performed with weighted UniFrac analysis with clustering at 97% sequence identity.

The rarefaction curves for NS-Perio and S-Perio groups stabilized at >1,500 OTUs with 14,000 sequences, showing greater bacterial diversity than that observed for NS-Control (950 OTUs). Regarding the two periodontal groups, the S-Perio group had a lower bacterial diversity than that found for the NS-Perio group (approximately 1,600 OTUs vs. 2,000 OTUs) (Figure 1A). These results were corroborated by Chao richness and Shannon diversity indexes for the different groups (4,892 and 5.32 for NS-Perio, 3,745 and 5.16 for S-Perio, 1,925 and 4.04 for NS-Control).

When the Principal Coordinates Analysis (PCoA) was performed with all 82 patients separately, this showed that subjects from each study group tended to cluster together, occupying a different position in the 3D space. However, an intragroup variation was detected in the S-Perio group, as evidenced by the overlap between many samples from S-Perio and NS-Perio. By two-way comparisons in microbial composition, we found statistically significant differences between three study groups (UniFrac distance, *p* ≤ 0.001 in all groups) (Figure 1B).

#### Taxonomy

The average, standard deviation, and range of sequences per sample were 2,541, 4,235, 691-19,219 (NS-Control); 1,932, 2,516, 613-12,475 (NS-Perio); 2,389, 3,073, 520-14,805 (S-Perio). The percentages of unclassified sequences in each study group ranged from 3.32 to 7.74%.

In all samples, 115 bacterial genera were identified, 68, 64, and 47% of them being present in more than half of controls and periodontal patients, respectively. However, there was a high inter-individual variability in the relative abundance at bacterial genera in both controls and periodontal patients (Figure 1 in Supplementary Material).

The genera present in 90–100% of the patients were *Fusobacterium, Parvimonas, Prevotella, Porphyromonas, Streptococcus*, and *TM7 genera incertae sedis* (Table 1 in Supplementary Material). The most abundant genus in the microbiota of all patients was *Fusobacterium* (47.79% in NS-Control, 32.00% in NS-Perio, and 36.65% in S-Perio) (Table 2 in Supplementary Material). Within Fusobacteria, the most abundant species was *Fusobacterium nucleatum* in the NS-Control and *Fusobacterium simiae* in periodontal patients (Table 3 in Supplementary Material).

**Figure 2:**
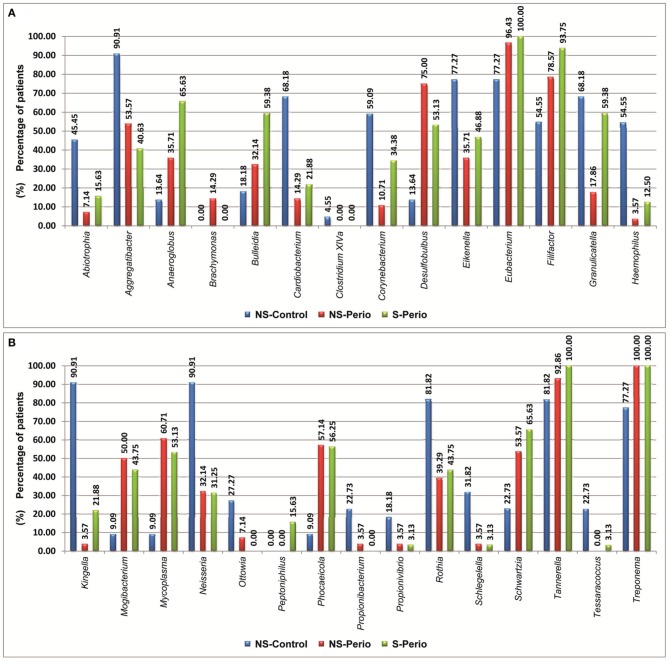
**Prevalence of patients being positive for different genera, which presented statistically significant differences between three study groups. (A)** Genera shown by alphabetical order: A–H. **(B)** Genus shown by alphabetical order: K–T.

**Figure 3:**
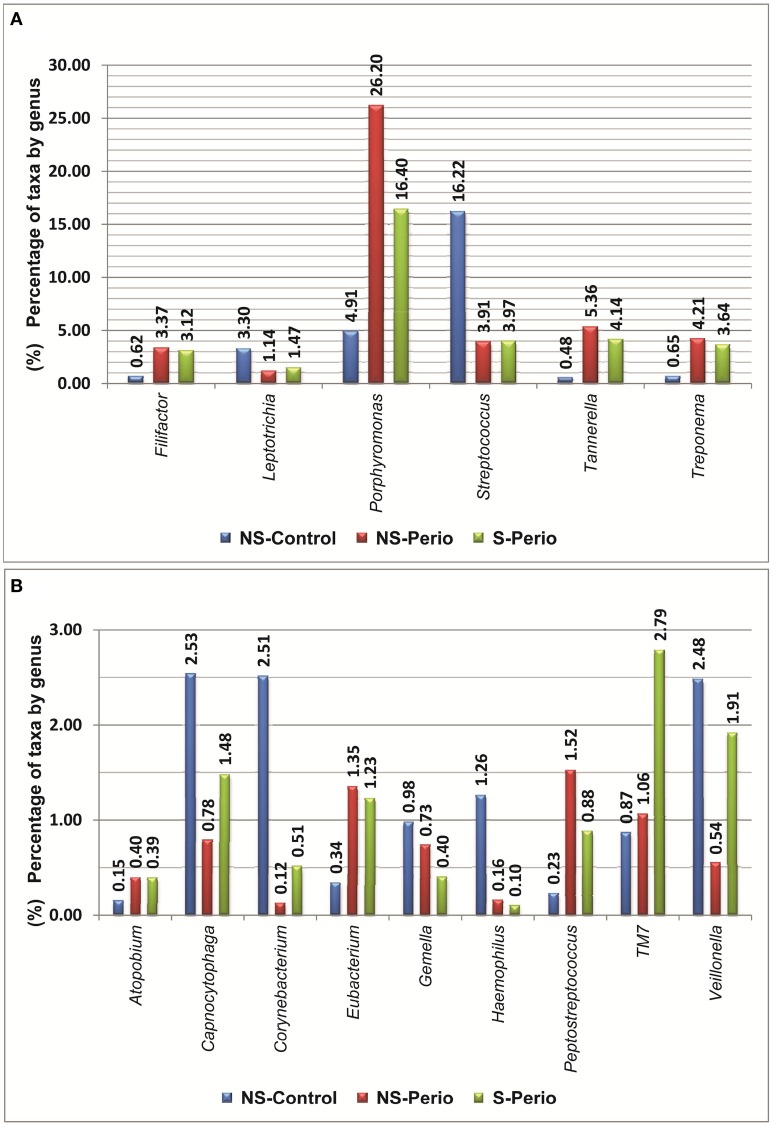
**Percentages of taxa by genera, which presented statistically significant differences were detected between three study groups. (A)** Percentages above 3% at least one study group. **(B)** Percentages below 3% in all study groups.

In comparison with the periodontal groups, the NS-Control showed a higher prevalence of patients being positive for 12 genera (Figure 2). NS-Control had higher abundance percentages of *Capnocytophaga*, *Corynebacterium*, *Gemella, Haemophilus, Leptotrichia, Streptococcus* and *Veillonella* (Figure 3). Sixteen health-associated species (with a positive higher difference than 20% between controls and either periodontal groups) were identified, which are detailed in Table 2.

**Table 2:** **Identified species associated with the health or chronic periodontitis, which showed a positive difference equal to or higher than 10% (in terms of relative abundance) between three study groups**.

**Differences between 10 and 20%^a^**		
**NS-Control vs. NS-Perio**	**NS-Perio vs. NS-Control**	**S-Perio vs. NS-Control**
*Leptotrichia goodfellowii*	*Capnocytophaga sputigena*	*Neisseria flavescens*
*Leptotrichia hofstadii*	*Neisseria flavescens*	*Neisseria iguanae*
	*Prevotella intermedia*	*Streptococcus gordonii*
	*Streptococcus mitis*	*Streptococcus mitis*
**NS-Control vs. S-Perio**	**NS-Perio vs. S-Perio**	**S-Perio vs. NS-Perio**
*Leptotrichia trevisanii*	*Eubacterium nodatum*	*Haemophilus felis*
*Neisseria elongata*	*Leptotrichia buccalis*	*Haemophilus influenzae*
	*Leptotrichia goodfellowii*	*Leptotrichia hofstadii*
	*Neisseria elongata*	
**Differences than >20%^a^**		
**NS-Control vs. NS-Perio**	**NS-Perio vs. NS-Control**	**S-Perio vs. NS-Control**
*Capnocytophaga ochracea*	*Bulleidia extructa*	*Bulleidia extructa*
*Eubacterium saburreum*	*Desulfobulbus propionicus*	*Capnocytophaga leadbetteri*
*Fusobacterium nucleatum*	*Eubacterium nodatum*	*Cardiobacterium hominis*
*Haemophilus aegyptius*	*Eubacterium saphenum*	*Desulfobulbus propionicus*
*Haemophilus influenzae*	*Fusobacterium simiae*	*Eubacterium infirmum*
*Kingella oralis*	*Kingella denitrificans*	*Eubacterium saphenum*
*Mogibacterium vescum*	*Leptotrichia wadei*	*Fusobacterium simiae*
*Mycoplasma salivarium*	*Mycoplasma faucium*	*Gemella haemolysans*
*Neisseria canis*	*Neisseria shayeganii*	*Haemophilus aegyptius*
*Porphyromonas catoniae*	*Phocaeicola abscessus*	*Kingella denitrificans*
*Prevotella oris*	*Porphyromonas gingivalis*	*Leptotrichia wadei*
*Propionibacterium acnes*	*Rothia mucilaginosa*	*Mycoplasma faucium*
*Propionivibrio pelophilus*	*Treponema denticola*	*Phocaeicola abscessus*
*Rothia dentocariosa*	*Treponema medium*	*Porphyromonas gingivalis*
*Streptococcus sanguinis*		*Rothia mucilaginosa*
*Treponema socranskii*		*Treponema denticola*
		*Treponema medium*
**NS-Control vs. S-Perio**	**NS-Perio vs. S-Perio**	**S-Perio vs. NS-Perio**
*Capnocytophaga ochracea*	*Capnocytophaga sputigena*	*Capnocytophaga leadbetteri*
*Cardiobacterium valvarum*	*Cardiobacterium valvarum*	*Cardiobacterium hominis*
*Eubacterium saburreum*	*Eubacterium saphenum*	*Eubacterium infirmum*
*Fusobacterium nucleatum*	*Fusobacterium nucleatum*	*Fusobacterium simiae*
*Gemella morbillorum*	*Gemella morbillorum*	*Gemella haemolysans*
*Haemophilus influenzae*	*Neisseria shayeganii*	*Haemophilus aegyptius*
*Kingella oralis*		*Mogibacterium vescum*
*Leptotrichia goodfellowii*		*Neisseria canis*
*Mycoplasma salivarium*		*Neisseria iguanae*
*Neisseria canis*		*Propionivibrio pelophilus*
*Porphyromonas catoniae*		
*Prevotella oris*		
*Propionibacterium acnes*		
*Rothia dentocariosa*		
*Streptococcus sanguinis*		
*Treponema socranskii*		

*NS-Control, Group of non-smoker healthy controls; NS-Perio, Group of non-smoker periodontal patients; S-Perio, Group of smoker periodontal patients*.

a *Selected genera for identification at the species-level were: Abiotrophia, Aggregatibacter, Anaeroglobus, Atopobium, Brachymonas, Bulleidia, Capnocytophaga, Cardiobacterium, Clostridium XIVa, Corynebacterium, Desulfobulbus, Eikenella, Eubacterium, Filifactor, Fusobacterium, Gemella, Granulicatella, Haemophilus, Kingella, Leptotrichia, Micoplasma, Mogibacterium, Neisseria, Ottowia, Parvimonas, Peptoniphilus, Peptostreptococcus, Phocaeicola, Porphyromonas, Prevotella, Propionibacterium, Propionivibrio, Rothia, Schlegelella, Schwartzia, Streptococcus, Tannerella, Tessaracoccus, Treponema, and Veillonella*.

In comparison with the NS-Control, the NS-Perio, and S-Perio groups showed a higher prevalence of patients being positive for 11 genera (Figure 2). The periodontal groups had higher abundance percentages of *Atopobium, Eubacterium, Filifactor, Peptostreptococcus, Porphyromonas, Tannerella, TM7*, and *Treponema* (Figure 3). Fourteen and 17 periodontitis-associated species in NS-Perio and S-Perio groups, respectively (with a positive higher difference than 20% between either periodontal groups and controls) were identified, which are detailed in Table 2.

With respect to the influence of smoking, *Anaeroglobus*, *Bulleidia (B. extructa)*, *Corynebacterium (C. durum)* and *Granulicatella* (*G. adiacens*) were more prevalent in the S-Perio group than in the NS-Perio group (after correction for multiple testing, the difference was only significant for *Granulicatella; G. adiacens*, 59.38 vs. 17.86%). On the other hand, *Porphyromonas* showed a significantly higher abundance in the NS-Perio group than that obtained in the S-Perio group (26 vs. 16%) while *Veillonella* and *TM7* had significantly lower percentages (after correction for multiple testing, the difference was only significant for *TM7*; 1.06 vs. 2.78%). Higher differences than 20% in the relative abundance between NS-Perio group and S-Perio group were detected in 16 species, which are detailed in Table 2.

A significant positive correlation in the relative abundance was found between *Filifactor*, *Eubacterium*, *Peptostreptococcus* (especially in NS-Control and NS-Perio groups), *Porphyromonas*, *Tannerella*, and *Treponema*, while a significant negative correlation was observed among *Capnocytophaga, Fusobacterium, Streptococcus*, and *Veillonella* with respect to *Filifactor*, *Porphyromonas*, *Tannerella*, and *Treponema* (Table 4 in Supplementary Material). In NS-Control and S-Perio groups, unlike NS-Perio, a significant positive correlation was observed between *TM7* with respect to *Eubacterium, Porphyromonas*, and *Tannerella*.

### Microbiota composition and clinical parameters: influence of smoking habit

**Figure 4:**
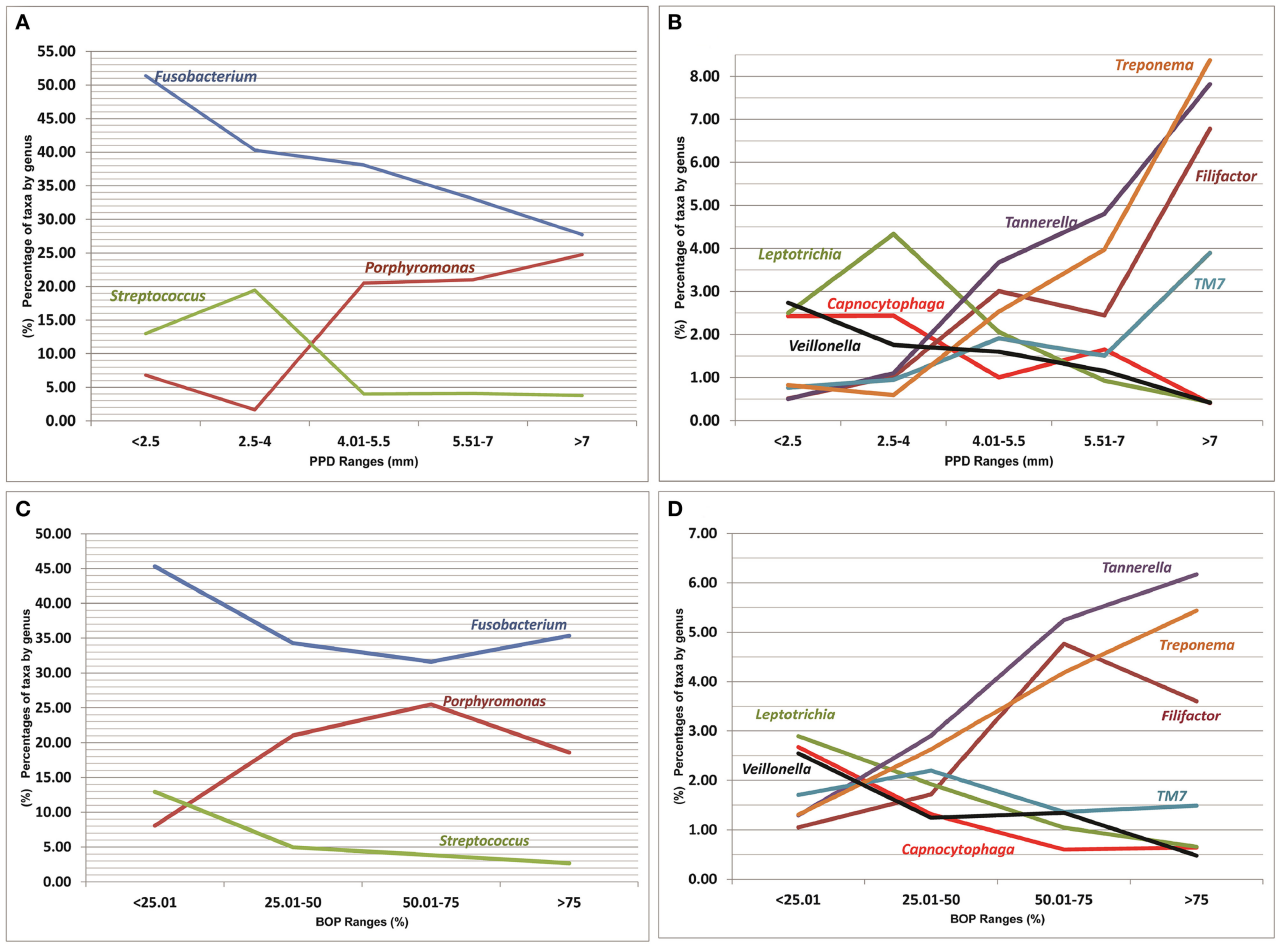
**Relationships between percentages of more significant genera (present in ≥50% of patients), represented as a percentage of the total, and values of clinical parameters. (A,B)** Probing pocket depth of sampled sites (mm). **(C,D)** Bleeding on probing of sampled sites (percentage). A mean percentage of taxa by genus was calculated for each PPD and BOP range.

A list of bacterial genera that were significantly correlated with different health/disease-associated clinical parameters is shown in Figure 4 (a full list is shown in Table 4 in Supplementary Material). The genera identified as *Capnocytophaga*, *Corynebacterium (C. durum)*, *Fusobacterium* (mainly *F. nucleatum*), *Gemella*, *Haemophilus*, *Leptotrichia, Streptococcus*, and *Veillonella* (especially in NS-Control and NS-Perio groups) were significantly negatively correlated with PPD, CAL, and/or BOP. *Eubacterium*, *Filifactor* (*F. alocis*; especially in NS-Control and NS-Perio groups), *Peptostreptococcus* (*P. stomatis*), *Porphyromonas* (mainly *P. gingivalis*), *Tannerella* (*T. forsythia*), and *Treponema* (mainly *T. denticola* and *T. medium*) were significantly positively correlated with one or more of these clinical parameters. In NS-Control and S-Perio groups, the genus *TM7* was significantly positively correlated with PPD, CAL, and BOP.

## Discussion

The current study evaluates the composition of the subgingival microbiota in health and chronic periodontitis, considering the influence smoking on disease. A major drawback of previous studies was their low sample size (Liu et al., 2012; Bizzarro et al., 2013) or low sequence coverage (Kumar et al., 2005). Thus, the relatively large groups of patients included in the current work, and the use of high-throughput sequencing, allowed the determination of the periodontitis-associated subgingival microbiota and its relationship with smoking and clinical parameters, confirming results from a statistical point of view; these analysis being extremely rare in studies published previously.

It has been considered that a more diverse bacterial community represents a more stable and healthy ecosystem (Griffen et al., 2012). In fact, oral diseases such as caries have been associated with a decrease in bacterial diversity (Jiang et al., 2014).

In accordance with previous authors (Griffen et al., 2012; Abusleme et al., 2013), in the present study, bacterial diversity (in both richness and evenness) was higher in patients with chronic periodontitis than in healthy subjects. However, in disagreement with the results reported by Bizzarro et al. (2013), bacterial diversity in periodontal patients decreased with smoking habit.

From a clinical point view, frequently the non-smoking periodontal patients show significantly higher values of BOP than those detected in the smoking periodontal patients, which was also observed in the present series. In our opinion, it would be very interesting to analyze the influence of this clinical parameter on the differences detected in the bacterial diversity of subgingival microbiota between non-smoking periodontal patients and smoking periodontal patients.

In addition, the PCoA showed a significantly different microbial structure between health, non-smoking-associated periodontitis and smoking-associated periodontitis. These results agree with those described by Kistler et al. (2013), who compared health vs. periodontitis, and by Ge et al. (2013), who observed that the differences in microbiome structure between deep and shallow sites in periodontal patients revealed by cluster analysis was influenced by patient-level effects such as smoking. Also, our data show it is important to distinguish taxa at the species-level, as different species within the same genus may be either health-associated and disease-associated species; this was observed in 13 bacterial genera (for example, *Porphyromonas catoniae* was a health-associated species while *Porphyromonas gingivalis* was disease-associated; a full list is shown in Table 2).

Our results corroborate that *Fusobacterium* species, especially *F. nucleatum*, have an important function in the subgingival biofilm, probably due to its bridging function among microorganisms, which allows attachment of periodontal bacteria (Signat et al., 2011). The majority of the health-associated species identified in the present series have been previously associated with oral health conditions by other authors using the Human Oral Microbe Identification Microarray (HOMIM) or 16S pyrosequencing (Colombo et al., 2009; Kistler et al., 2013). Interestingly, although the genus *Streptococcus* was equally represented in health and periodontitis (present in all subjects), the proportions of *Streptococcus sanguinis* were increased in health while others such as *Streptococcus mitis* were associated with disease.

Although *Porphyromonas* was present in virtually all subjects, its relative abundance was higher in those with chronic periodontitis. However, *Tannerella* and *Treponema* showed higher values in periodontal patients in both, presence and relative abundance. We confirm prior findings, obtained using low-throughput techniques (Socransky et al., 1998) and highly paralleled pyrosequencing (Scher et al., 2012; Abusleme et al., 2013), which suggested that the advanced forms of periodontitis display an overrepresentation of the species *P. gingivalis, T. forsythia* and *T. denticola* (“red complex” species; recently termed “keystone pathogens”), all of which have been implicated in the pathogenesis of chronic periodontitis (Socransky et al., 1998). Agreeing with Li et al. (2014), in the present study, there was no difference in the presence/relative abundance in the genus *Prevotella* between healthy individuals and periodontal patients (a total of 22 species were identified; further details in Table 3 in Supplementary Material). However, *Prevotella intermedia* was more abundant in the periodontal patients (23.67 and 14.07 vs. 4.79% in NS-Control), confirming its involvement as a member of the “orange complex” in the development of the chronic periodontitis (Socransky et al., 1998). Of note all “established” periopathogens were found (although at low levels) in healthy individuals showing these organisms are not acting as exogenous pathogens.

Our results revealed also the colonization by novel bacteria associated with periodontitis that clearly deserve more attention such as *Desulfobulbus (D. propionicus)*, *Mogibacterium* (*M. vescum*), *Phocaeicola (P. abscessus*), and *Filifactor (F. alocis*). *D. propionicus* was six times more prevalent in periodontal patients than in healthy subjects. *Desulfobulbus* has been previously identified in periodontitis (Bizzarro et al., 2013) and related to a poor response to periodontal therapy (Colombo et al., 2009). In the present study, *F. alocis* appeared to be strongly disease-associated and positively co-associated with “established” periopathogens such as *Porphyromonas*, *Tannerella*, and *Treponema*. Aruni et al. (2011) showed that *F. alocis* has virulence properties that may favor its ability to survive and persist in the periodontal pocket and how these virulence attributes were enhanced in coculture with *P. gingivalis*. Recently, Fine et al. (2013) observed a synergistic interaction of the association between *A. actinomycetemcomitans*, *S. parasanguinis*, and *F. alocis* and how this consortium indicated sites of future bone loss in the localized aggressive periodontitis. We are the first to report an association of *M. vescum* and *P. abscessus* with chronic periodontitis (in terms of presence). Equally, although *Leptotrichia* and *Rothia* were health-associated genera, we found a higher abundance of *Leptotrichia wadei* and *Rothia mucilaginosa* in the periodontal patients. This suggests that more knowledge about “unusual” species involved in the chronic periodontitis is required.

There are only a few published manuscripts on the comparison of the subgingival microbiota in smokers and non-smokers with chronic periodontitis applying NGS methodologies. Agreeing with Bizzarro et al. (2013), our findings showed the microbial community of smoking-related periodontitis to be distinct from that of non-smokers. These authors detected a higher proportion of *Fusobacterium*, and novel genera such as *Paludibacter* and *Desulfobulbus* in periodontal patients, especially smokers. However, in the present study, *Anaeroglobus* and *Bulleidia (B. extructa)* were seven and five times more prevalent in smoking-associated periodontitis than in health, respectively. There is no literature on the implication of these bacterial genera in the pathogenesis of chronic periodontitis (especially in smoker patients). We are also the first to detect a three-fold increase in relative abundance of the *TM7* candidate division in smoker periodontal patients with respect non-smokers and a co-association with “well-known” periopathogens. This bacterial group deserves further study, as it has been related to the inflammatory pathogenesis of periodontitis (Brinig et al., 2003; Ouverney et al., 2003).

Based on their own findings, Hajishengallis et al. (2011) confirmed the importance of the identification and targeting of low-abundance pathogens for diagnosis and treating inflammatory diseases of polymicrobial etiology. Colombo et al. (2009) suggested, based on the results obtained by Human Oral Microbe Identification Microarray technique, that a greater presence and abundance of an “unusual” subgingival microbiota could explain the poor response of subjects with refractory periodontitis to conventional periodontal therapy combined with systemic antibiotics. However, to date, the specific role that these novel low-abundance genera and species (some of them, especially associated with smoking) play in the initiation and/or progression of chronic periodontitis is unknown. On the other hand, it has been demonstrated that smoking can directly affect the host's ability to control the infection and as a consequence, elimination or control of the microbiota, especially “unusual” species, would be more difficult, increasing the risk for treatment failure (Johannsen et al., 2014).

Our results on the positive correlation between the “established” periopathogens and the clinical parameters corroborate those previously published by Socransky et al. (1998), who applied whole genomic DNA probes and checkerboard DNA–DNA hybridization and observed that the “red complex” species were strongly related to pocket depth and bleeding on probing. However, we are the first to report a positive co-association of “unusual” genera with these clinical parameters, such as *Filifactor*, and the influence of smoking habit (the *TM7* group deserves a special mention in smoking periodontal patients with respect to the controls, where it was significantly positively correlated with PPD, CAL, and BOP). An intriguing finding detected in the present work was the negative relationship of *Fusobacterium* with these clinical parameters, especially pocket depth. Although it has been recognized that *F. nucleatum* has periodontopathogenic properties (Signat et al., 2011), our results raise doubts on its role as “commensal” or “pathogen.”

Hajishengallis and Lamont (2012) proposed a new model of periodontal pathogenesis according in which periodontitis is initiated by a synergistic and dysbiotic microbial community rather than by select “periopathogens,” such as the “red complex” (PSD model). Our findings support this hypothesis and show that chronic periodontitis is caused by a dysbiotic change in the periodontal microbiota, in other words a change in the presence/relative abundance of individual members of the microbiota. These include “established” periopathogens and a number of other novel taxa, all of which are better referred to as “pathobionts” (Simón-Soro and Mira, 2015). This polymicrobial synergy and dysbiosis could be responsible for changes in the complex host–microbe interactions sufficient to initiate chronic inflammatory disease.

On the other hand, the microbial community of smoking-associated periodontitis is less diverse and distinct from that of non-smokers, indicating that smoking has an influence on periodontal ecology.

Future studies including serial samples from the same subjects (longitudinal study design) will improve our understanding of periodontal pathogenesis on the basis of the PSD model, identifying keystone and accessory pathogens (instead of “pathogens” and “commensals”) and promoting the development of novel therapeutic strategies. In this sense, we anticipate that therapies directed toward red-complex periodontal pathogens alone may not be effective.

### Conflict of interest statement

The authors declare that the research was conducted in the absence of any commercial or financial relationships that could be construed as a potential conflict of interest.
