# Supplementary material for: Subgingival microbiota in health compared to periodontitis and the influence of smoking
Source: Front Microbiol. 2015 Feb 24;6:119. doi: 10.3389/fmicb.2015.00119 (PMC4356944; doi:10.3389/fmicb.2015.00119)
Supplement: Supplementary file 1 [file Table1.DOCX]

**Table 1**. Number of subjects and percentage of them (between parenthesis), as well as statistical significance between three study groups.

| **Genus** | **STUDY GROUPS (n)** | | | | | | | | | |
| --- | --- | --- | --- | --- | --- | --- | --- | --- | --- | --- |
|  | **NS-Control (I group; n=22)** | | **NS-Perio (II group; n=28)** | | **S-Perio (III group; n=32)** | | **P value**  **(between all grous)^1^** | **Significant pairwise comparisons^2^** | | |
| *Abiotrophia* | 10 | (45.45) | 2 | (7.14) | 5 | (15.63) | 0.009 | I-II | I-III* |  |
| *Acinetobacter* | 0 | (0.00) | 1 | (3.57) | 0 | (0.00) | NS |  |  |  |
| *Actinobacillus* | 2 | (9.09) | 0 | (0.00) | 0 | (0.00) | NS |  |  |  |
| *Actinomyces* | 14 | (63.64) | 13 | (46.43) | 19 | (59.38) | NS |  |  |  |
| *Aggregatibacter* | 20 | (90.91) | 15 | (53.57) | 13 | (40.63) | <0.001 | I-II | I-III |  |
| *Alkalibacterium* | 1 | (4.55) | 0 | (0.00) | 1 | (3.13) | NS |  |  |  |
| *Anaerobacillus* | 1 | (4.55) | 0 | (0.00) | 1 | (3.13) | NS |  |  |  |
| *Anaeroglobus* | 3 | (13.64) | 10 | (35.71) | 21 | (65.63) | <0.001 |  | I-III | II-III* |
| *Anaerotruncus* | 0 | (0.00) | 0 | (0.00) | 1 | (3.13) | NS |  |  |  |
| *Anaerovorax* | 0 | (0.00) | 0 | (0.00) | 1 | (3.13) | NS |  |  |  |
| *Atopobium* | 9 | (40.91) | 15 | (53.57) | 19 | (59.38) | NS |  |  |  |
| *Bacteroides* | 0 | (0.00) | 5 | (17.86) | 7 | (21.88) | NS |  |  |  |
| *Barnesiella* | 2 | (9.09) | 0 | (0.00) | 0 | (0.00) | NS |  |  |  |
| *Bergeyella* | 2 | (9.09) | 0 | (0.00) | 0 | (0.00) | NS |  |  |  |
| *Blautia* | 0 | (0.00) | 0 | (0.00) | 2 | (6.25) | NS |  |  |  |
| *Brachymonas* | 0 | (0.00) | 4 | (14.29) | 0 | (0.00) | 0.040 |  |  | II-III* |
| *Bulleidia* | 4 | (18.18) | 9 | (32.14) | 19 | (59.38) | 0.007 |  | I-III | II-III* |
| *Burkholderia* | 1 | (4.55) | 2 | (7.14) | 5 | (15.63) | NS |  |  |  |
| *Butyrivibrio* | 0 | (0.00) | 3 | (10.71) | 1 | (3.13) | NS |  |  |  |
| *Campylobacter* | 20 | (90.91) | 25 | (89.29) | 26 | (81.25) | NS |  |  |  |
| *Capnocytophaga* | 22 | (100.00) | 21 | (75.00) | 26 | (81.25) | NS |  |  |  |
| *Cardiobacterium* | 15 | (68.18) | 4 | (14.29) | 7 | (21.88) | <0.001 | I-II | I-III |  |
| *Carnobacterium* | 1 | (4.55) | 0 | (0.00) | 2 | (6.25) | NS |  |  |  |
| *Catenibacterium* | 0 | (0.00) | 0 | (0.00) | 1 | (3.13) | NS |  |  |  |
| *Catonella* | 10 | (45.45) | 18 | (64.29) | 18 | (56.25) | NS |  |  |  |
| *Cellulomonas* | 0 | (0.00) | 0 | (0.00) | 1 | (3.13) | NS |  |  |  |
| *Centipeda* | 2 | (9.09) | 3 | (10.71) | 5 | (15.63) | NS |  |  |  |
| *Clostridium XI* | 0 | (0.00) | 0 | (0.00) | 1 | (3.13) | NS |  |  |  |
| *Clostridium XIX* | 10 | (45.45) | 16 | (57.14) | 22 | (68.75) | NS |  |  |  |
| *Clostridium XlVa* | 1 | (4.55) | 0 | (0.00) | 0 | (0.00) | 0.040 |  |  |  |
| *Collinsella* | 0 | (0.00) | 0 | (0.00) | 1 | (3.13) | NS |  |  |  |
| *Corynebacterium* | 13 | (59.09) | 3 | (10.71) | 11 | (34.38) | 0.001 | I-II |  | II-III* |
| *Craurococcus* | 0 | (0.00) | 1 | (3.57) | 0 | (0.00) | NS |  |  |  |
| *Desulfobulbus* | 3 | (13.64) | 21 | (75.00) | 17 | (53.13) | <0.001 | I-II | I-III |  |
| *Desulfomicrobium* | 0 | (0.00) | 0 | (0.00) | 1 | (3.13) | NS |  |  |  |
| *Dialister* | 16 | (72.73) | 20 | (71.43) | 28 | (87.50) | NS |  |  |  |
| *Dolosigranulum* | 0 | (0.00) | 0 | (0.00) | 1 | (3.13) | NS |  |  |  |
| *Eikenella* | 17 | (77.27) | 10 | (35.71) | 15 | (46.88) | 0.026 | I-II | I-III* |  |
| *Enterococcus* | 2 | (9.09) | 0 | (0.00) | 2 | (6.25) | NS |  |  |  |
| *Erysipelotrichaceae*  *_incertae_sedis* | 1 | (4.55) | 0 | (0.00) | 2 | (6.25) | NS |  |  |  |
| *Escherichia/Shigella* | 3 | (13.64) | 1 | (3.57) | 0 | (0.00) | NS |  |  |  |
| *Eubacterium* | 17 | (77.27) | 27 | (96.43) | 32 | (100.00) | <0.001 |  | I-III |  |
| *Exiguobacterium* | 0 | (0.00) | 0 | (0.00) | 1 | (3.13) | NS |  |  |  |
| *Faecalibacterium* | 2 | (9.09) | 0 | (0.00) | 0 | (0.00) | NS |  |  |  |
| *Filifactor* | 12 | (54.55) | 22 | (78.57) | 30 | (93.75) | 0.006 |  | I-III |  |
| *Fusobacterium* | 22 | (100.00) | 28 | (100.00) | 32 | (100.00) | NA |  |  |  |
| *Gemella* | 21 | (95.45) | 23 | (82.14) | 24 | (75.00) | NS |  |  |  |
| *Granulicatella* | 15 | (68.18) | 5 | (17.86) | 19 | (59.38) | <0.001 | I-II |  | II-III |
| *Haemophilus* | 12 | (54.5) | 1 | (3.57) | 4 | (12.50) | <0.001 | I-II | I-III |  |
| *Hafnia* | 0 | (0.00) | 2 | (7.14) | 1 | (3.13) | NS |  |  |  |
| *Hallella* | 4 | (18.18) | 14 | (50.00) | 12 | (37.50) | NS |  |  |  |
| *Hydrogenophaga* | 1 | (4.55) | 0 | (0.00) | 0 | (0.00) | NS |  |  |  |
| *Johnsonella* | 9 | (40.91) | 12 | (42.86) | 12 | (37.50) | NS |  |  |  |
| *Ketogulonicigenium* | 0 | (0.00) | 0 | (0.00) | 0 | (0.00) | NS |  |  |  |
| *Kingella* | 20 | (90.91) | 1 | (3.57) | 7 | (21.88) | <0.001 | I-II | I-III |  |
| *Lactobacillus* | 1 | (4.55) | 0 | (0.00) | 3 | (9.38) | NS |  |  |  |
| *Lactococcus* | 0 | (0.00) | 1 | (3.57) | 0 | (0.00) | NS |  |  |  |
| *Leptotrichia* | 21 | (95.45) | 25 | (89.29) | 28 | (87.50) | NS |  |  |  |
| *Leuconostoc* | 1 | (4.55) | 0 | (0.00) | 2 | (6.25) | NS |  |  |  |
| *Maritalea* | 0 | (0.00) | 0 | (0.00) | 1 | (3.13) | NS |  |  |  |
| *Megasphaera* | 2 | (9.09) | 2 | (7.14) | 6 | (18.7) | NS |  |  |  |
| *Micrococcus* | 1 | (4.55) | 0 | (0.00) | 0 | (0.00) | NS |  |  |  |
| *Mogibacterium* | 2 | (9.09) | 14 | (50.00) | 14 | (43.75) | 0.005 | I-II | I-III |  |
| *Moryella* | 3 | (13.64) | 10 | (35.71) | 14 | (43.75) | NS |  |  |  |
| *Murdochiella* | 0 | (0.00) | 0 | (0.00) | 1 | (3.13) | NS |  |  |  |
| *Mycoplasma* | 2 | (9.09) | 17 | (60.71) | 17 | (53.13) | <0.001 | I-II | I-III |  |
| *Neisseria* | 20 | (90.91) | 9 | (32.14) | 10 | (31.25) | <0.001 | I-II | I-III |  |
| *Obesumbacterium* | 0 | (0.00) | 0 | (0.00) | 1 | (3.13) | NS |  |  |  |
| *Odoribacter* | 0 | (0.00) | 2 | (7.14) | 7 | (21.88) | NS |  |  |  |
| *Oribacterium* | 6 | (27.27) | 6 | (21.43) | 7 | (21.88) | NS |  |  |  |
| *Ottowia* | 6 | (27.27) | 2 | (7.14) | 0 | (0.00) | 0.006 |  | I-III |  |
| *Paracoccus* | 2 | (9.09) | 0 | (0.00) | 1 | (3.13) | NS |  |  |  |
| *Paraprevotella* | 0 | (0.00) | 0 | (0.00) | 1 | (3.13) | NS |  |  |  |
| *Parvimonas* | 20 | (90.91) | 27 | (96.43) | 32 | (100.00) | NS |  |  |  |
| *Peptoniphilus* | 0 | (0.00) | 0 | (0.00) | 5 | (15.63) | 0.031 |  |  |  |
| *Peptostreptococcaceae*  *_incertae_sedis* | 12 | (54.55) | 12 | (42.86) | 12 | (37.50) | NS |  |  |  |
| *Peptostreptococcus* | 8 | (36.36) | 18 | (64.29) | 21 | (65.63) | NS |  |  |  |
| *Phocaeicola* | 2 | (9.09) | 16 | (57.14) | 18 | (56.25) | <0.001 | I-II | I-III |  |
| *Pilibacter* | 0 | (0.00) | 0 | (0.00) | 1 | (3.13) | NS |  |  |  |
| *Porphyromonas* | 22 | (100.00) | 28 | (100.00) | 32 | (100.00) | NA |  |  |  |
| *Prevotella* | 22 | (100.00) | 28 | (100.00) | 32 | (100.00) | NA |  |  |  |
| *Propionibacterium* | 5 | (22.73) | 1 | (3.57) | 0 | (0.00) | 0.010 |  | I-III |  |
| *Propionivibrio* | 4 | (18.18) | 1 | (3.57) | 1 | (3.13) | 0.039 |  |  |  |
| *Pseudomonas* | 3 | (13.64) | 6 | (21.43) | 7 | (21.88) | NS |  |  |  |
| *Pseudoramibacter* | 2 | (9.09) | 3 | (10.71) | 6 | (18.75) | NS |  |  |  |
| *Psychrobacter* | 1 | (4.55) | 0 | (0.00) | 0 | (0.00) | NS |  |  |  |
| *Pyramidobacter* | 0 | (0.00) | 4 | (14.29) | 6 | (18.75) | NS |  |  |  |
| *Ralstonia* | 1 | (4.55) | 0 | (0.00) | 1 | (3.13) | NS |  |  |  |
| *Rhodococcus* | 0 | (0.00) | 0 | (0.00) | 1 | (3.13) | NS |  |  |  |
| *Rothia* | 18 | (81.82) | 11 | (39.29) | 14 | (43.75) | 0.007 | I-II | I-III |  |
| *Scardovia* | 0 | (0.00) | 1 | (3.57) | 1 | (3.13) | NS |  |  |  |
| *Schlegelella* | 7 | (31.82) | 1 | (3.57) | 1 | (3.13) | 0.004 | I-II | I-III |  |
| *Schwartzia* | 5 | (22.73) | 15 | (53.57) | 21 | (65.63) | 0.010 | I-II* | I-III |  |
| *Selenomonas* | 17 | (77.27) | 26 | (92.86) | 28 | (87.50) | NS |  |  |  |
| *Serratia* | 1 | (4.55) | 3 | (10.71) | 4 | (12.50) | NS |  |  |  |
| *Shuttleworthia* | 1 | (4.55) | 3 | (10.71) | 7 | (21.88) | NS |  |  |  |
| *Sneathia* | 2 | (9.09) | 6 | (21.43) | 8 | (25.00) | NS |  |  |  |
| *Solobacterium* | 10 | (45.45) | 15 | (53.57) | 20 | (62.50) | NS |  |  |  |
| *Sphingobium* | 1 | (4.55) | 0 | (0.00) | 0 | (0.00) | NS |  |  |  |
| *Sphingomonas* | 2 | (9.09) | 0 | (0.00) | 0 | (0.00) | NS |  |  |  |
| *Sporanaerobacter* | 1 | (4.55) | 0 | (0.00) | 0 | (0.00) | NS |  |  |  |
| *Sporolactobacillaceae*  *_incertae_sedis* | 0 | (0.00) | 0 | (0.00) | 1 | (3.13) | NS |  |  |  |
| *SR1_genera*  *incertae_sedis* | 8 | (36.36) | 10 | (35.71) | 11 | (34.38) | NS |  |  |  |
| *Staphylococcus* | 3 | (13.64) | 0 | (0.00) | 1 | (3.13) | NS |  |  |  |
| *Streptococcus* | 22 | (100.00) | 28 | (100.00) | 31 | (96.88) | NS |  |  |  |
| *Streptophyta* | 1 | (4.55) | 0 | (0.00) | 0 | (0.00) | NS |  |  |  |
| *Synergistes* | 0 | (0.00) | 0 | (0.00) | 1 | (3.13) | NS |  |  |  |
| *Tannerella* | 18 | (81.82) | 26 | (92.86) | 32 | (100.00) | 0.039 |  | I-III* |  |
| *Tessaracoccus* | 5 | (22.73) | 0 | (0.00) | 1 | (3.13) | 0.012 | I-II | I-III* |  |
| *TM7_genera*  *incertae_sedis* | 21 | (95.45) | 26 | (92.86) | 29 | (90.63) | NS |  |  |  |
| *Treponema* | 17 | (77.27) | 28 | (100.00) | 32 | (100.00) | 0.003 | I-II | I-III |  |
| *Turicibacter* | 0 | (0.00) | 0 | (0.00) | 1 | (3.13) | NS |  |  |  |
| *Veillonella* | 21 | (95.45) | 23 | (82.14) | 26 | (81.25) | NS |  |  |  |
| *Weissella* | 1 | (4.55) | 0 | (0.00) | 0 | (0.00) | NS |  |  |  |
| *Wolinella* | 0 | (0.00) | 1 | (3.57) | 2 | (6.25) | NS |  |  |  |
| *Xylanibacter* | 0 | (0.00) | 0 | (0.00) | 1 | (3.13) | NS |  |  |  |

NS-Control= Group of non-smoker healthy controls; NS-Perio= Group of non-smoker periodontal patients; S-Perio= Group of smoker periodontal patients; NS= not significant.

1- Statistical differences between all study groups were analyzed using the Fisher’s exact test with p<0.05.

2**-** Statistical differences between two groups were analyzed using the Fisher’s exact test with p<0.05.

*After Bonferroni correction for multiple analyses (the significance level applied was p< 0.016), the differences were no longer significant for these comparisons.
