# Supplementary material for: Subgingival microbiota in health compared to periodontitis and the influence of smoking
Source: Front Microbiol. 2015 Feb 24;6:119. doi: 10.3389/fmicb.2015.00119 (PMC4356944; doi:10.3389/fmicb.2015.00119)
Supplement: Supplementary file 2 [file Table2.DOCX]

**Table 2**. Mean percentage of taxa by genus, with standard deviation (between parenthesis), as well as statistical significance between 3 study groups.

| **Genus*** | **STUDY GROUPS (n)** | | | | | | | | | |
| --- | --- | --- | --- | --- | --- | --- | --- | --- | --- | --- |
|  | **NS-Control (I group; n=22)** | | **NS-Perio (II group; n=28)** | | **S-Perio (III group; n=32)** | | **P Value**  **(between all grous)^1^** | **Significant pairwise comparisons^2^** | | |
| *Abiotrophia* | 0.5062 | (0.6504) | 0.0545 | (0.0254) | 0.2550 | (0.3599) | NS |  |  |  |
| *Acinetobacter* | 0.0000 | (0.0000) | 0.2183 | (0.0000) | 0.0000 | (0.0000) | NA |  |  |  |
| *Actinobacillus* | 0.1168 | (0.0272) | 0.0000 | (0.0000) | 0.0000 | (0.0000) | NA |  |  |  |
| *Actinomyces* | 1.0034 | (1.4721) | 0.1872 | (0.2032) | 0.5915 | (1.4129) | NS |  |  |  |
| *Aggregatibacter* | 1.0306 | (1.7339) | 0.3612 | (0.2913) | 0.3000 | (0.2509) | NS |  |  |  |
| *Alkalibacterium* | 0.0976 | (0.0000) | 0.0000 | (0.0000) | 0.0472 | (0.0000) | NS |  |  |  |
| *Anaerobacillus* | 0.4545 | (0.0000) | 0.0000 | (0.0000) | 0.0472 | (0.0000) | NS |  |  |  |
| *Anaeroglobus* | 0.1050 | (0.0852) | 0.1511 | (0.0929) | 0.4065 | (0.4163) | NS |  |  |  |
| *Anaerotruncus* | 0.0000 | (0.0000) | 0.0000 | (0.0000) | 0.1565 | (0.0000) | NA |  |  |  |
| *Anaerovorax* | 0.0000 | (0.0000) | 0.0000 | (0.0000) | 0.0357 | (0.0000) | NA |  |  |  |
| *Atopobium* | 0.1509 | (0.1851) | 0.3970 | (0.5176) | 0.3923 | (0.3502) | 0.048 |  | I-III |  |
| *Bacteroides* | 0.0000 | (0.0000) | 0.0933 | (0.0365) | 0.3317 | (0.6743) | NS |  |  |  |
| *Barnesiella* | 0.2951 | (0.3363) | 0.0000 | (0.0000) | 0.0000 | (0.0000) | NA |  |  |  |
| *Bergeyella* | 0.0765 | (0.0977) | 0.0000 | (0.0000) | 0.0000 | (0.0000) | NA |  |  |  |
| *Blautia* | 0.0000 | (0.0000) | 0.0000 | (0.0000) | 0.2796 | (0.3384) | NA |  |  |  |
| *Brachymonas* | 0.0000 | (0.0000) | 0.1004 | (0.0995) | 0.0000 | (0.0000) | NA |  |  |  |
| *Bulleidia* | 0.0490 | (0.0387) | 0.3256 | (0.3929) | 0.2848 | (0.3452) | NS |  |  |  |
| *Burkholderia* | 1.3780 | (0.0000) | 11.5166 | (13.1705) | 0.2297 | (0.2301) | NS |  |  |  |
| *Butyrivibrio* | 0.0000 | (0.0000) | 0.2852 | (0.2808) | 0.0832 | (0.0000) | NS |  |  |  |
| *Campylobacter* | 0.6184 | (0.5719) | 0.4507 | (0.3068) | 0.5257 | (0.4707) | NS |  |  |  |
| *Capnocytophaga* | 2.5332 | (2.4374) | 0.7846 | (0.7290) | 1.4786 | (3.4073) | 0.002 | I-II | I-III |  |
| *Cardiobacterium* | 0.2300 | (0.3237) | 0.1407 | (0.0939) | 0.0748 | (0.0586) | NS |  |  |  |
| *Carnobacterium* | 0.1050 | (0.0000) | 0.0000 | (0.0000) | 4.8342 | (6.7699) | NS |  |  |  |
| *Catenibacterium* | 0.0000 | (0.0000) | 0.0000 | (0.0000) | 0.0472 | (0.0000) | NA |  |  |  |
| *Catonella* | 0.2561 | (0.2499) | 0.2396 | (0.1816) | 0.1664 | (0.1171) | NS |  |  |  |
| *Cellulomonas* | 0.0000 | (0.0000) | 0.0000 | (0.0000) | 0.0472 | (0.0000) | NA |  |  |  |
| *Centipeda* | 0.0331 | (0.0178) | 0.0850 | (0.0451) | 0.0387 | (0.0244) | NS |  |  |  |
| *Clostridium XI* | 0.0000 | (0.0000) | 0.0000 | (0.0000) | 0.8019 | (0.0000) | NA |  |  |  |
| *Clostridium XIX* | 0.1952 | (0.1599) | 0.1949 | (0.0938) | 0.2254 | (0.1672) | NS |  |  |  |
| *Clostridium XlVa* | 0.1776 | (0.0000) | 0.0000 | (0.0000) | 0.0000 | (0.0000) | NS |  |  |  |
| *Collinsella* | 0.0000 | (0.0000) | 0.0000 | (0.0000) | 0.1415 | (0.0000) | NA |  |  |  |
| *Corynebacterium* | 2.5118 | (4.5071) | 0.1226 | (0.1064) | 0.5123 | (0.7099) | 0.022 | I-II* | I-III* |  |
| *Craurococcus* | 0.0000 | (0.0000) | 0.1258 | (0.0000) | 0.0000 | (0.0000) | NS |  |  |  |
| *Desulfobulbus* | 0.1355 | (0.1357) | 0.7034 | (0.5150) | 0.7373 | (0.9331) | NS |  |  |  |
| *Desulfomicrobium* | 0.0000 | (0.0000) | 0.0000 | (0.0000) | 0.6623 | (0.0000) | NA |  |  |  |
| *Dialister* | 0.4727 | (0.6239) | 0.4591 | (0.4513) | 0.6566 | (0.8098) | NS |  |  |  |
| *Dolosigranulum* | 0.0000 | (0.0000) | 0.0000 | (0.0000) | 0.0867 | (0.0000) | NA |  |  |  |
| *Eikenella* | 0.2436 | (0.1695) | 0.1681 | (0.1530) | 0.1945 | (0.1692) | NS |  |  |  |
| *Enterococcus* | 0.2977 | (0.2818) | 0.0000 | (0.0000) | 0.2575 | (0.1694) | NS |  |  |  |
| *Erysipelotrichaceae*  *incertae_sedis* | 0.0438 | (0.0000) | 0.0000 | (0.0000) | 0.2130 | (0.2345) | NS |  |  |  |
| *Escherichia/Shigella* | 0.1187 | (0.0692) | 0.0803 | (0.0000) | 0.0000 | (0.0000) | NS |  |  |  |
| *Eubacterium* | 0.3396 | (0.3040) | 1.3500 | (1.3828) | 1.2305 | (1.1704) | 0.000 | I-II | I-III |  |
| *Exiguobacterium* | 0.0000 | (0.0000) | 0.0000 | (0.0000) | 0.1415 | (0.0000) | NA |  |  |  |
| *Faecalibacterium* | 0.1195 | (0.1070) | 0.0000 | (0.0000) | 0.0000 | (0.0000) | NA |  |  |  |
| *Filifactor* | 0.6225 | (0.7267) | 3.3659 | (3.6556) | 3.1153 | (3.9874) | 0.001 | I-II | I-III |  |
| *Fusobacterium* | 47.7950 | (25.9869) | 32.0028 | (17.8137) | 36.6500 | (21.3810) | NS |  |  |  |
| *Gemella* | 0.9785 | (1.0092) | 0.7320 | (0.8304) | 0.4025 | (0.5610) | 0.024 |  | I-III |  |
| *Granulicatella* | 1.2754 | (3.8472) | 0.2428 | (0.1946) | 0.2640 | (0.3329) | NS |  |  |  |
| *Haemophilus* | 1.2590 | (2.1899) | 0.1550 | (0.0000) | 0.0975 | (0.0760) | 0.030 |  | I-III |  |
| *Hafnia* | 0.0000 | (0.0000) | 0.1532 | (0.0026) | 65.6566 | (0.0000) | NS |  |  |  |
| *Hallella* | 0.1072 | (0.0482) | 0.4506 | (0.3565) | 0.3259 | (0.3348) | NS |  |  |  |
| *Hydrogenophaga* | 0.0210 | (0.0000) | 0.0000 | (0.0000) | 0.0000 | (0.0000) | NA |  |  |  |
| *Johnsonella* | 0.2684 | (0.1524) | 0.1294 | (0.0912) | 0.1752 | (0.1873) | NS |  |  |  |
| *Ketogulonicigenium* | 0.0000 | (0.0000) | 0.0000 | (0.0000) | 0.0000 | (0.0000) | NA |  |  |  |
| *Kingella* | 0.6040 | (0.8775) | 0.0803 | (0.0000) | 0.4562 | (0.8896) | NS |  |  |  |
| *Lactobacillus* | 0.1645 | (0.0000) | 0.0000 | (0.0000) | 0.2655 | (0.2295) | NS |  |  |  |
| *Lactococcus* | 0.0000 | (0.0000) | 0.3101 | (0.0000) | 0.0000 | (0.0000) | NA |  |  |  |
| *Leptotrichia* | 3.3015 | (4.1854) | 1.1400 | (1.5609) | 1.4728 | (2.4418) | 0.013 | I-II | I-III* |  |
| *Leuconostoc* | 0.1145 | (0.0000) | 0.0000 | (0.0000) | 0.3283 | (0.2500) | NS |  |  |  |
| *Maritalea* | 0.0000 | (0.0000) | 0.0000 | (0.0000) | 0.1031 | (0.0000) | NA |  |  |  |
| *Megasphaera* | 0.1750 | (0.2178) | 0.1480 | (0.0526) | 0.3271 | (0.3256) | NS |  |  |  |
| *Micrococcus* | 0.0069 | (0.0000) | 0.0000 | (0.0000) | 0.0000 | (0.0000) | NA |  |  |  |
| *Mogibacterium* | 0.0140 | (0.0093) | 0.1552 | (0.2125) | 0.1607 | (0.1908) | NS |  |  |  |
| *Moryella* | 0.0732 | (0.0555) | 0.1225 | (0.0536) | 0.0936 | (0.0564) | NS |  |  |  |
| *Murdochiella* | 0.0000 | (0.0000) | 0.0000 | (0.0000) | 0.2013 | (0.0000) | NA |  |  |  |
| *Mycoplasma* | 0.1939 | (0.1910) | 0.3882 | (0.3901) | 0.2682 | (0.3169) | NS |  |  |  |
| *Neisseria* | 1.2557 | (1.5438) | 0.2994 | (0.2282) | 0.4024 | (0.3059) | NS |  |  |  |
| *Obesumbacterium* | 0.0000 | (0.0000) | 0.0000 | (0.0000) | 0.2525 | (0.0000) | NA |  |  |  |
| *Odoribacter* | 0.0000 | (0.0000) | 0.9722 | (0.9205) | 1.5373 | (1.5540) | NS |  |  |  |
| *Oribacterium* | 0.1411 | (0.1071) | 0.1202 | (0.0610) | 0.1189 | (0.0975) | NS |  |  |  |
| *Ottowia* | 0.3225 | (0.3178) | 0.1239 | (0.0026) | 0.0000 | (0.0000) | NS |  |  |  |
| *Paracoccus* | 0.1470 | (0.0433) | 0.0000 | (0.0000) | 0.0472 | (0.0000) | NS |  |  |  |
| *Paraprevotella* | 0.0000 | (0.0000) | 0.0000 | (0.0000) | 0.0120 | (0.0000) | NA |  |  |  |
| *Parvimonas* | 1.3743 | (1.3430) | 1.2994 | (1.2041) | 1.6457 | (1.7625) | NS |  |  |  |
| *Peptoniphilus* | 0.0000 | (0.0000) | 0.0000 | (0.0000) | 0.1459 | (0.0684) | NA |  |  |  |
| *Peptostreptococcaceae*  *incertae_sedis* | 0.4902 | (0.3416) | 0.4387 | (0.9443) | 0.2520 | (0.2135) | NS |  |  |  |
| *Peptostreptococcus* | 0.2272 | (0.2917) | 1.5248 | (1.4682) | 0.8815 | (1.0137) | 0.013 | I-II | I-III* |  |
| *Phocaeicola* | 0.0532 | (0.0058) | 0.2636 | (0.2457) | 0.4981 | (0.6322) | NS |  |  |  |
| *Pilibacter* | 0.0000 | (0.0000) | 0.0000 | (0.0000) | 0.0357 | (0.0000) | NA |  |  |  |
| *Porphyromonas* | 4.9116 | (7.4306) | 26.1969 | (14.7989) | 16.3993 | (14.4392) | 0.000 | I-II | I-III | II-III |
| *Prevotella* | 7.7163 | (8.2024) | 8.3252 | (7.7563) | 10.1959 | (8.3894) | NS |  |  |  |
| *Propionibacterium* | 0.0800 | (0.0684) | 0.1185 | (0.0000) | 0.0000 | (0.0000) | NS |  |  |  |
| *Propionivibrio* | 0.1647 | (0.1121) | 0.0803 | (0.0000) | 0.1894 | (0.0000) | NS |  |  |  |
| *Pseudomonas* | 2.7839 | (4.6167) | 6.6404 | (9.5833) | 4.7401 | (7.2985) | NS |  |  |  |
| *Pseudoramibacter* | 0.0326 | (0.0139) | 0.0450 | (0.0241) | 0.0804 | (0.0868) | NS |  |  |  |
| *Psychrobacter* | 0.1242 | (0.0000) | 0.0000 | (0.0000) | 0.0000 | (0.0000) | NA |  |  |  |
| *Pyramidobacter* | 0.0000 | (0.0000) | 0.3650 | (0.2783) | 0.3363 | (0.5130) | NS |  |  |  |
| *Ralstonia* | 0.0424 | (0.0000) | 0.0000 | (0.0000) | 0.1894 | (0.0000) | NS |  |  |  |
| *Rhodococcus* | 0.0000 | (0.0000) | 0.0000 | (0.0000) | 1.0281 | (0.0000) | NA |  |  |  |
| *Rothia* | 0.4554 | (0.4642) | 0.1893 | (0.1363) | 0.2492 | (0.2167) | NS |  |  |  |
| *Scardovia* | 0.0000 | (0.0000) | 0.2516 | (0.0000) | 0.1786 | (0.0000) | NS |  |  |  |
| *Schlegelella* | 0.5742 | (0.7975) | 0.0417 | (0.0000) | 0.0251 | (0.0000) | NS |  |  |  |
| *Schwartzia* | 0.1472 | (0.1800) | 0.3047 | (0.2970) | 0.1979 | (0.1430) | NS |  |  |  |
| *Selenomonas* | 0.6497 | (0.8556) | 0.6273 | (0.5639) | 0.5408 | (0.5198) | NS |  |  |  |
| *Serratia* | 0.0210 | (0.0000) | 14.4495 | (14.8910) | 10.8150 | (21.3862) | NS |  |  |  |
| *Shuttleworthia* | 0.0147 | (0.0000) | 0.2395 | (0.2217) | 0.2567 | (0.3346) | NS |  |  |  |
| *Sneathia* | 0.0586 | (0.0506) | 8.8739 | (10.2614) | 3.3809 | (6.5575) | NS |  |  |  |
| *Solobacterium* | 0.3157 | (0.3262) | 0.5226 | (0.6998) | 0.3632 | (0.5332) | NS |  |  |  |
| *Sphingobium* | 0.1456 | (0.0000) | 0.0000 | (0.0000) | 0.0000 | (0.0000) | NA |  |  |  |
| *Sphingomonas* | 0.1376 | (0.0566) | 0.0000 | (0.0000) | 0.0000 | (0.0000) | NA |  |  |  |
| *Sporanaerobacter* | 0.1717 | (0.0000) | 0.0000 | (0.0000) | 0.0000 | (0.0000) | NA |  |  |  |
| *Sporolactobacillaceae*  *incertae_sedis* | 0.0000 | (0.0000) | 0.0000 | (0.0000) | 0.0472 | (0.0000) | NA |  |  |  |
| *SR1_genera*  *incertae_sedis* | 0.1579 | (0.1373) | 0.1320 | (0.0636) | 0.2440 | (0.3037) | NS |  |  |  |
| *Staphylococcus* | 0.2171 | (0.1220) | 0.0000 | (0.0000) | 0.0403 | (0.0000) | NS |  |  |  |
| *Streptococcus* | 16.2192 | (15.4659) | 3.9076 | (3.4652) | 3.9723 | (3.9470) | 0.000 | I-II | I-III |  |
| *Streptophyta* | 0.0274 | (0.0000) | 0.0000 | (0.0000) | 0.0000 | (0.0000) | NA |  |  |  |
| *Synergistes* | 0.0000 | (0.0000) | 0.0000 | (0.0000) | 0.1721 | (0.0000) | NA |  |  |  |
| *Tannerella* | 0.4814 | (0.4105) | 5.3567 | (3.1064) | 4.1398 | (3.7616) | 0.000 | I-II | I-III |  |
| *Tessaracoccus* | 0.0660 | (0.0459) | 0.0000 | (0.0000) | 0.0472 | (0.0000) | NS |  |  |  |
| *TM7_genera*  *incertae_sedis* | 0.8663 | (0.8087) | 1.0616 | (1.0072) | 2.7856 | (3.3072) | 0.005 |  | I-III | II-III |
| *Treponema* | 0.6535 | (0.7941) | 4.2107 | (3.7354) | 3.6353 | (3.2663) | 0.000 | I-II | I-III |  |
| *Turicibacter* | 0.0000 | (0.0000) | 0.0000 | (0.0000) | 0.3774 | (0.0000) | NS |  |  |  |
| *Veillonella* | 2.4796 | (3.3177) | 0.5422 | (0.7203) | 1.9128 | (2.8082) | 0.003 | I-II |  | II-III* |
| *Weissella* | 0.0572 | (0.0000) | 0.0000 | (0.0000) | 0.0000 | (0.0000) | NA |  |  |  |
| *Wolinella* | 0.0000 | (0.0000) | 0.0730 | (0.0000) | 0.0635 | (0.0559) | NA |  |  |  |
| *Xylanibacter* | 0.0000 | (0.0000) | 0.0000 | (0.0000) | 0.0456 | (0.0000) | NA |  |  |  |

NS-Control= Group of non-smoker healthy controls; NS-Perio= Group of non-smoker periodontal patients; S-Perio= Group of smoker periodontal patients; NS= not significant; NA.

1- Statistical differences between all groups were analyzed by Kruskal-Wallis test with p<0.05

2**-** Statistical differences between 2 groups were analyzed by U Mann-Whitney test with p<0.05

*After Bonferroni correction for multiple analyses (the significance level applied was p< 0.016), the differences were no longer significant for these comparisons.
