# Supplementary material for: Subgingival microbiota in health compared to periodontitis and the influence of smoking
Source: Front Microbiol. 2015 Feb 24;6:119. doi: 10.3389/fmicb.2015.00119 (PMC4356944; doi:10.3389/fmicb.2015.00119)
Supplement: Supplementary file 3 [file Table3.DOCX]

**Table 3.** Percentage of sequences from each genus assigned to the different species available in public databases, in the three study groups.

| Genus^1^ | Species | NS-Control (%)^1^ | NS-Perio (%)^1^ | S-Perio (%)^1^ |
| --- | --- | --- | --- | --- |
| *Aggregatibacter* | *segnis* | 100.00 | 100.00 | 100.00 |
| *Atopobium* | *parvulum* | 17.02 | 15.29 | 10.44 |
| *Atopobium* | *rimae* | 82.98 | 84.71 | 89.56 |
| *Bulleidia* | *extructa* | 0.00 | 100.00 | 100.00 |
| *Capnocytophaga* | *gingivalis* | 11.79 | 16.99 | 11.13 |
| *Capnocytophaga* | *leadbetteri* | 41.52 | 46.33 | 74.20 |
| *Capnocytophaga* | *ochracea* | 37.94 | 15.25 | 13.98 |
| *Capnocytophaga* | *sputigena* | 8.75 | 21.43 | 0.69 |
| *Cardiobacterium* | *hominis* | 37.57 | 40.21 | 72.59 |
| *Cardiobacterium* | *valvarum* | 62.43 | 59.79 | 27.41 |
| *Corynebacterium* | *durum* | 100.00 | 100.00 | 100.00 |
| *Desulfobulbus* | *propionicus* | 0.00 | 100.00 | 100.00 |
| *Eikenella* | *corrodens* | 100.00 | 100.00 | 100.00 |
| *Eubacterium* | *infirmum* | 3.25 | 3.24 | 37.73 |
| *Eubacterium* | *minutum* | 0.00 | 0.88 | 6.41 |
| *Eubacterium* | *nodatum* | 0.00 | 21.10 | 5.16 |
| *Eubacterium* | *saburreum* | 89.24 | 9.19 | 17.40 |
| *Eubacterium* | *saphenum* | 7.52 | 65.59 | 33.30 |
| *Filifactor* | *alocis* | 100.00 | 100.00 | 100.00 |
| *Fusobacterium* | *nucleatum* | 88.95 | 64.69 | 40.29 |
| *Fusobacterium* | *russii* | 0.01 | 0.00 | 0.00 |
| *Fusobacterium* | *simiae* | 11.04 | 35.31 | 59.71 |
| *Gemella* | *haemolysans* | 33.01 | 28.82 | 54.37 |
| *Gemella* | *morbillorum* | 66.99 | 69.59 | 45.63 |
| *Gemella* | *sanguinis* | 0.00 | 1.59 | 0.00 |
| *Granulicatella* | *adiacens* | 100.00 | 100.00 | 100.00 |
| *Haemophilus* | *aegyptius* | 40.98 | 0.00 | 71.95 |
| *Haemophilus* | *ducreyi* | 6.03 | 0.00 | 0.00 |
| *Haemophilus* | *felis* | 5.99 | 0.00 | 13.69 |
| *Haemophilus* | *influenzae* | 41.64 | 0.00 | 14.36 |
| *Haemophilus* | *parasuis* | 5.36 | 0.00 | 0.00 |
| *Kingella* | *denitrificans* | 1.71 | 30.94 | 25.73 |
| *Kingella* | *oralis* | 98.29 | 69.06 | 74.27 |
| *Leptotrichia* | *buccalis* | 25.38 | 33.39 | 18.00 |
| *Leptotrichia* | *goodfellowii* | 24.24 | 12.30 | 1.29 |
| *Leptotrichia* | *hofstadii* | 27.73 | 8.90 | 28.35 |
| *Leptotrichia* | *hongkongensis* | 3.48 | 1.55 | 9.29 |
| *Leptotrichia* | *shahii* | 0.00 | 1.76 | 0.00 |
| *Leptotrichia* | *trevisanii* | 11.47 | 8.78 | 1.34 |
| *Leptotrichia* | *wadei* | 7.71 | 33.33 | 41.73 |
| *Mogibacterium* | *vescum* | 100.00 | 0.00 | 100.00 |
| *Mycoplasma* | *faucium* | 0.00 | 54.66 | 51.16 |
| *Mycoplasma* | *hyopharyngis* | 0.00 | 0.00 | 2.90 |
| *Mycoplasma* | *salivarium* | 100.00 | 45.34 | 45.94 |
| *Neisseria* | *bacilliformis* | 1.09 | 0.00 | 5.21 |
| *Neisseria* | *canis* | 57.71 | 3.22 | 32.73 |
| *Neisseria* | *elongata* | 21.44 | 22.41 | 10.29 |
| *Neisseria* | *flavescens* | 13.77 | 25.98 | 27.90 |
| *Neisseria* | *gonorrhoeae* | 0.37 | 0.00 | 1.08 |
| *Neisseria* | *iguanae* | 5.27 | 0.00 | 21.18 |
| *Neisseria* | *shayeganii* | 0.36 | 48.39 | 0.50 |
| *Neisseria* | *subflava* | 0.00 | 0.00 | 1.10 |
| *Parvimonas* | *micra* | 100.00 | 100.00 | 100.00 |
| *Peptostreptococcus* | *stomatis* | 100.00 | 100.00 | 100.00 |
| *Phocaeicola* | *abscessus* | 0.00 | 100.00 | 100.00 |
| *Porphyromonas* | *catoniae* | 36.73 | 0.73 | 4.56 |
| *Porphyromonas* | *gingivalis* | 63.27 | 99.27 | 95.44 |
| *Prevotella* | *baroniae* | 0.27 | 0.16 | 0.38 |
| *Prevotella* | *buccae* | 0.17 | 0.19 | 0.78 |
| *Prevotella* | *denticola* | 4.10 | 4.58 | 8.88 |
| *Prevotella* | *disiens* | 0.00 | 0.00 | 0.05 |
| *Prevotella* | *enoeca* | 0.00 | 0.08 | 0.39 |
| *Prevotella* | *fusca* | 0.00 | 0.15 | 0.26 |
| *Prevotella* | *intermedia* | 4.79 | 23.67 | 14.07 |
| *Prevotella* | *loescheii* | 5.02 | 6.52 | 4.97 |
| *Prevotella* | *maculosa* | 2.35 | 0.85 | 1.44 |
| *Prevotella* | *marshii* | 0.00 | 0.00 | 1.14 |
| *Prevotella* | *micans* | 0.00 | 3.16 | 0.70 |
| *Prevotella* | *multiformis* | 0.00 | 0.48 | 0.13 |
| *Prevotella* | *nigrescens* | 20.78 | 19.75 | 23.62 |
| *Prevotella* | *oralis* | 0.18 | 0.61 | 3.22 |
| *Prevotella* | *oris* | 42.90 | 22.39 | 14.53 |
| *Prevotella* | *oulorum* | 6.96 | 1.64 | 1.93 |
| *Prevotella* | *pallens* | 0.00 | 0.09 | 0.63 |
| *Prevotella* | *pleuritidis* | 3.02 | 3.09 | 10.83 |
| *Prevotella* | *scopos* | 0.00 | 0.14 | 0.20 |
| *Prevotella* | *shahii* | 0.00 | 0.00 | 3.20 |
| *Prevotella* | *tannerae* | 8.66 | 7.56 | 7.31 |
| *Prevotella* | *veroralis* | 0.81 | 4.90 | 1.34 |
| *Propionibacterium* | *acnes* | 100.00 | 0.00 | 0.00 |
| *Propionivibrio* | *pelophilus* | 100.00 | 0.00 | 100.00 |
| *Rothia* | *aeria* | 4.47 | 0.00 | 2.63 |
| *Rothia* | *dentocariosa* | 92.11 | 62.17 | 62.33 |
| *Rothia* | *mucilaginosa* | 3.42 | 37.83 | 35.03 |
| *Streptococcus* | *anginosus* | 0.67 | 10.18 | 3.11 |
| *Streptococcus* | *cristatus* | 26.40 | 26.62 | 25.25 |
| *Streptococcus* | *gordonii* | 2.22 | 6.92 | 13.94 |
| *Streptococcus* | *infantis* | 2.96 | 10.78 | 5.23 |
| *Streptococcus* | *lactarius* | 0.17 | 0.16 | 0.00 |
| *Streptococcus* | *massiliensis* | 0.09 | 0.17 | 0.00 |
| *Streptococcus* | *mitis* | 8.76 | 19.34 | 20.69 |
| *Streptococcus* | *mutans* | 0.31 | 0.00 | 0.12 |
| *Streptococcus* | *oligofermentans* | 0.14 | 0.53 | 0.00 |
| *Streptococcus* | *oralis* | 1.06 | 4.10 | 6.60 |
| *Streptococcus* | *peroris* | 0.39 | 0.60 | 0.25 |
| *Streptococcus* | *salivarius* | 0.38 | 0.00 | 0.25 |
| *Streptococcus* | *sanguinis* | 56.44 | 20.61 | 24.37 |
| *Streptococcus* | *sinensis* | 0.00 | 0.00 | 0.20 |
| *Tannerella* | *forsythia* | 100.00 | 100.00 | 100.00 |
| *Treponema* | *amylovorum* | 0.00 | 2.31 | 3.03 |
| *Treponema* | *denticola* | 13.79 | 61.57 | 54.57 |
| *Treponema* | *medium* | 12.80 | 34.39 | 33.10 |
| *Treponema* | *parvum* | 0.00 | 0.38 | 1.87 |
| *Treponema* | *socranskii* | 73.41 | 1.35 | 7.43 |
| *Veillonella* | *denticariosi* | 29.36 | 23.75 | 20.00 |
| *Veillonella* | *dispar* | 0.89 | 0.00 | 0.93 |
| *Veillonella* | *parvula* | 69.75 | 76.25 | 79.07 |

NS-Control= Group of non-smoker healthy controls; NS-Perio= Group of non-smoker periodontal patients; S-Perio= Group of smoker periodontal patients.

1-Values of 100% indicate that all reads from a given genus were assigned to a single species
