# Supplementary material for: Subgingival microbiota in health compared to periodontitis and the influence of smoking
Source: Front Microbiol. 2015 Feb 24;6:119. doi: 10.3389/fmicb.2015.00119 (PMC4356944; doi:10.3389/fmicb.2015.00119)
Supplement: Supplementary file 4 [file Table4.DOCX]

**Table 4.** Spearman Correlation Coeficients between mean distributions of the more significant genera, and between these genera and clinical parameters (PPD, CAL and BP).

| ***Correlations*** | ***Atopobium*** |  | ***Capnocytophaga*** |  | ***Corynebacterium*** |  | ***Eubacterium*** |  | ***Filifactor*** |  | ***Fusobacterium*** |  | ***Gemella*** |  | ***Haemophilus*** |  | ***Leptotrichia*** |  | ***Peptostreptococcus*** |  | ***Porphyromonas*** |  | ***Streptococcus*** |  | ***Tannerella*** |  | ***TM7_genera_ incertae_sedis*** |  | ***Treponema*** |  | ***Veillonella*** |  |
| --- | --- | --- | --- | --- | --- | --- | --- | --- | --- | --- | --- | --- | --- | --- | --- | --- | --- | --- | --- | --- | --- | --- | --- | --- | --- | --- | --- | --- | --- | --- | --- | --- |
| ***Atopobium*** |  |  |  |  |  |  |  |  |  |  |  |  |  |  |  |  |  |  | *0.58* | **** |  |  |  |  |  |  | *0.51* | **** |  |  |  |  |
| ***Capnocytophaga*** |  |  |  |  | *0.55* | **** |  |  | *-0.28* | *** |  |  | *0.33* | *** | *0.61* | *** | *0.49* | **** |  |  | *-0.26* | *** | *0.49* | **** | *-0.46* | **** |  |  | *-0.38* | **** |  |  |
| ***Corynebacterium*** |  |  | *0.55* | **** |  |  |  |  | *-0.53* | *** |  |  |  |  |  |  | *0.44* | *** |  |  |  |  | *0.56* | **** |  |  |  |  |  |  |  |  |
| ***Eubacterium*** |  |  |  |  |  |  |  |  | *0.39* | **** | *-0.33* | **** |  |  |  |  |  |  | *0.45* | **** | *0.34* | **** |  |  | *0.49* | **** |  |  | *0.55* | **** | *-0.26* | *** |
| ***Filifactor*** |  |  | *-0.28* | *** | *-0.53* | *** | *0.39* | **** |  |  | *-0.33* | **** |  |  |  |  |  |  |  |  | *0.38* | **** | *-0.25* | *** | *0.49* | **** |  |  | *0.58* | **** |  |  |
| ***Fusobacterium*** |  |  |  |  |  |  | *-0.33* | **** | *-0.33* | **** |  |  |  |  | *-0.79* | **** |  |  |  |  | *-0.58* | **** |  |  | *-0.49* | **** | *-0.30* | **** | *-0.51* | **** |  |  |
| ***Gemella*** |  |  | *0.33* | *** |  |  |  |  |  |  |  |  |  |  |  |  | *0.41* | **** |  |  |  |  | *0.53* | **** | *-0.34* | **** |  |  |  |  | *0.29* | *** |
| ***Haemophilus*** |  |  | *0.61* | *** |  |  |  |  |  |  | *-0.79* | **** |  |  |  |  |  |  |  |  |  |  | *0.74* | **** | *-0.55* | *** |  |  |  |  |  |  |
| ***Leptotrichia*** |  |  | *0.49* | **** | *0.44* | *** |  |  |  |  |  |  | *0.41* | **** |  |  |  |  |  |  | *-0.23* | *** | *0.56* | **** | *-0.36* | **** | *0.27* | *** |  |  | *0.41* | **** |
| ***Peptostreptococcus*** | *0.58* | **** |  |  |  |  | *0.45* | **** |  |  |  |  |  |  |  |  |  |  |  |  |  |  |  |  | *0.41* | **** |  |  | *0.36* | *** |  |  |
| ***Porphyromonas*** |  |  | *-0.26* | *** |  |  | *0.34* | **** | *0.38* | **** | *-0.58* | **** |  |  |  |  | *-0.23* | *** |  |  |  |  | *-0.29* | **** | *0.66* | **** |  |  | *0.59* | **** | *-0.25* | *** |
| ***Streptococcus*** |  |  | *0.49* | **** | *0.56* | **** |  |  | *-0.25* | *** |  |  | *0.53* | **** | *0.74* | **** | *0.56* | **** |  |  | *-0.29* | **** |  |  | *-0.50* | **** | *0.31* | **** | *-0.36* | **** | *0.41* | **** |
| ***Tannerella*** |  |  | *-0.46* | **** | *-0.41* | *** | *0.49* | **** | *0.49* | **** | *-0.49* | **** | *-0.34* | **** | *-0.55* | *** | *-0.36* | **** | *0.41* | **** | *0.66* | **** | *-0.50* | **** |  |  |  |  | *0.78* | **** | *-0.40* | **** |
| ***TM7_genera_ incertae_sedis*** | *0.58* | **** |  |  |  |  |  |  |  |  | *-0.30* | **** |  |  |  |  | *0.27* | *** |  |  |  |  | *0.31* | **** |  |  |  |  |  |  |  |  |
| ***Treponema*** |  |  | *-0.38* | **** | *-0.50* | *** | *0.55* | **** | *0.58* | **** | *-0.51* | **** |  |  |  |  |  |  | *0.36* | *** | *0.59* | **** | *-0.36* | **** | *0.78* | **** |  |  |  |  | *-0.32* | **** |
| ***Veillonella*** |  |  |  |  |  |  | *-0.26* | *** |  |  |  |  | *0.29* | *** |  |  | *0.41* | **** |  |  | *-0.25* | *** | *0.41* | **** | *-0.40* | **** |  |  | *-0.32* | **** |  |  |
| ***PPD (mm)*** |  |  | *-0.46* | **** | *-0.50* | **** | *0.37* | **** | *0.31* | *** | *-0.23* | *** | *-0.27* | *** | *-0.52* | *** | *-0.40* | **** |  |  | *0.49* | **** | *-0.34* | **** | *0.56* | **** |  |  | *0.56* | **** | *-0.27* | *** |
| ***CAL (mm)*** |  |  | *-0.42* | **** | *-0.48* | *** | *0.33* | **** | *0.29* | *** |  |  | *-0.30* | *** | *-0.53* | *** | *-0.41* | **** |  |  | *0.43* | **** | *-0.32* | **** | *0.40* | **** | *0.24* | *** | *0.48* | **** |  |  |
| ***BOP (%)*** |  |  | *-0.44* | **** |  |  | *0.39* | **** | *0.38* | **** |  |  |  |  |  |  | *-0.31* | **** | *0.44* | **** | *0.42* | **** | *-0.40* | **** | *0.62* | **** |  |  | *0.59* | **** | *-0.37* | **** |

PPD= Probing pocket depth; CAL= Clinical attachment level; BOP= Bleeding on probing.

Significant positive correlations are shown in light green (rho <0.5) and dark green (rho ≥0.5). Significant positive correlations are shown in pink (rho <0.5) and red (rho ≥0.5).

(*) Significant correlation at level 0.05. (**) Significant correlation at level 0.01.
