# Supplementary figures and images for: Subgingival microbiota in health compared to periodontitis and the influence of smoking
Source: Front Microbiol. 2015 Feb 24;6:119. doi: 10.3389/fmicb.2015.00119 (PMC4356944; doi:10.3389/fmicb.2015.00119)

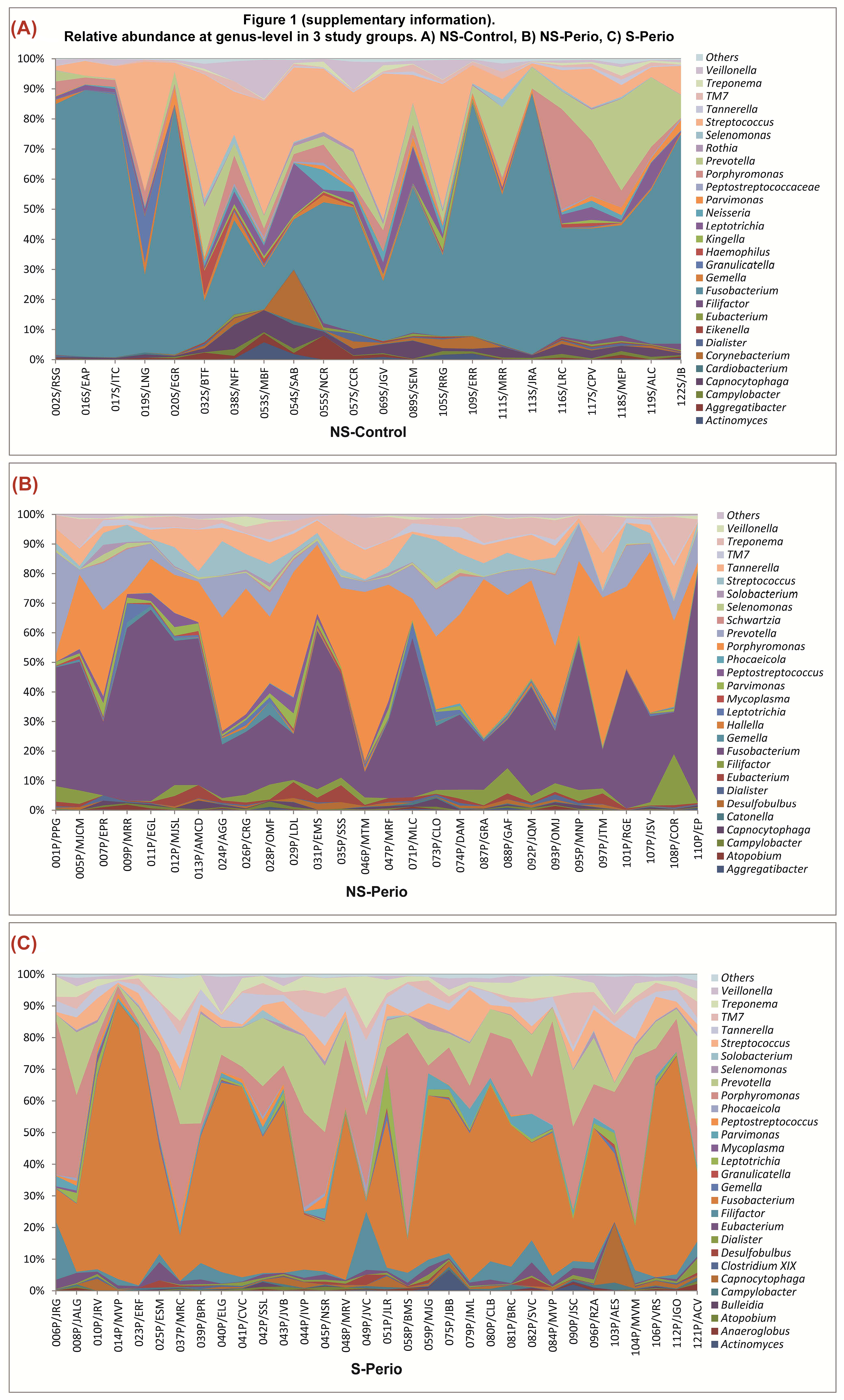

Supplement: Supplementary file 5 [file Image1.TIF]
